# 3-Aryl-2*H*-azirines as annulation reagents in the Ni(II)-catalyzed synthesis of 1*H*-benzo[4,5]thieno[3,2-*b*]pyrroles

**DOI:** 10.3762/bjoc.21.123

**Published:** 2025-08-11

**Authors:** Julia I Pavlenko, Pavel A Sakharov, Anastasiya V Agafonova, Derenik A Isadzhanyan, Alexander F Khlebnikov, Mikhail S Novikov

**Affiliations:** 1 Saint Petersburg State University, Institute of Chemistry, 7/9 Universitetskaya Naberezhnaya, St. Petersburg 199034, Russia

**Keywords:** annulation, azirines, benzothiophenes, indoles, nickel catalysis

## Abstract

A high-yielding method for the synthesis of 3-arylbenzo[4,5]thieno[3,2-*b*]pyrroles has been developed via pyrrole ring annulation to the aromatic benzo[*b*]thiophene system, using 3-arylazirines as a N‒C=C synthon. The reaction is catalyzed by Ni(hfacac)_2_ and proceeds through the azirine ring opening across the N=C3 bond. Azirines with both electron-donating and electron-withdrawing C3-aryl substituents tolerate the reaction conditions. The reaction of the *N*-methylindole analog also provides the annulation product but in moderate yield. The described synthesis is the first example of a dealkoxycarbonylative annulation reaction, in which 2*H*-azirines act as the annulation reagent.

## Introduction

**Scheme 1:**
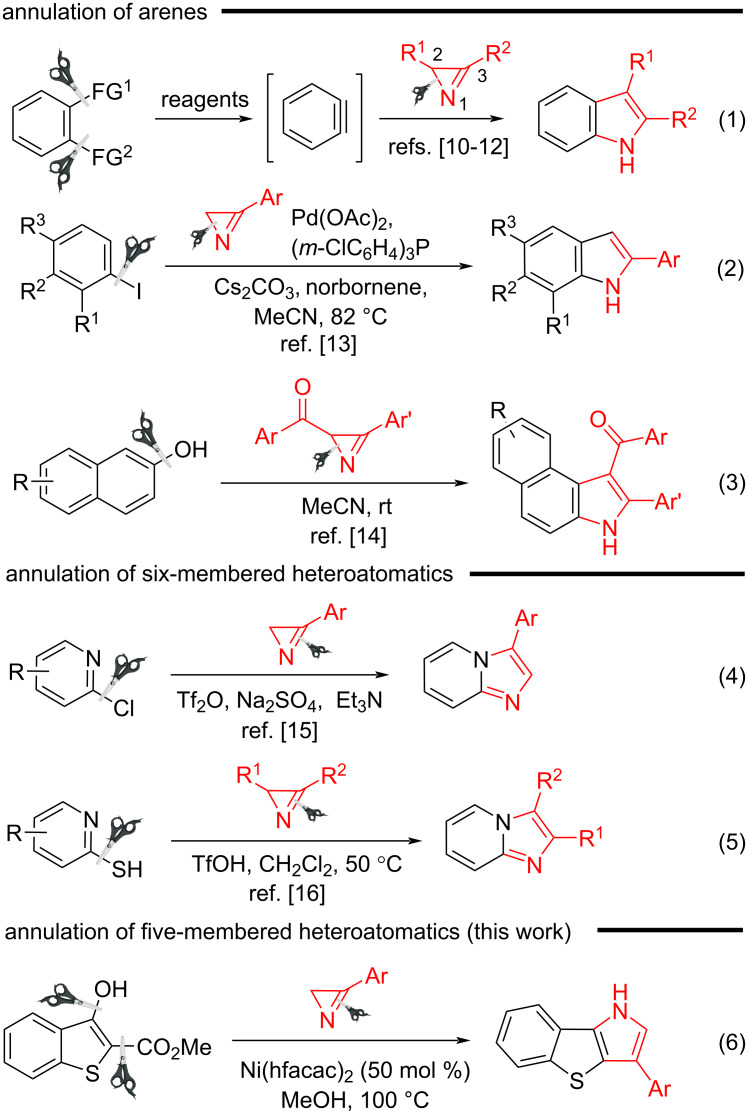
Synthesis of fused pyrroles and azoles by [3 + 2] annulation reactions of azirines.

2*H*-Azirines represent a valuable class of nitrogen heterocycles that are widely used as versatile building blocks in organic synthesis. In particular, the unique ability of these compounds to undergo selective opening of the three-membered ring at either of the two nitrogen–carbon bonds under certain conditions and to be incorporated into the target molecule as a N‒C‒C synthon is successfully used to obtain a variety of heterocyclic and acyclic nitrogen-containing compounds [1–3]. Such reactions can be initiated by electrophilic, nucleophilic or radical reagents, photoirradiation or proceed under acid-, metal-, or photocatalytic conditions. This strategy of azirine ring expansion is applicable to the synthesis of a variety of 4‒9-membered N-heterocycles, differing in the nature and number of heteroatoms and their mutual arrangement. These also include *ortho*-fused heterocycles, which are obtained from functionalized cyclic systems and azirines as annulation reagents. Significant progress has been achieved in the synthesis of pyrrolo-fused systems by the reactions of intermolecular annulation of a variety of non-aromatic five- [4–6] and six-membered carbo- and heterocycles [7–9] with azirines, which occur under transition metal catalysis or photocatalysis. Annulation of the pyrrole ring to an aromatic system is limited to reactions of functionalized arenes with azirines. These include the [3 + 2] cycloaddition of azirines to arynes (Scheme 1, reaction 1) [10–12], Pd-catalyzed reaction of azirines with iodoarenes (Scheme 1, reaction 2) [13], and reaction of 2-aroylazirines with naphthols (Scheme 1, reaction 3) [8]. The [3 + 2] annulation reactions of heteroaromatics with azirines are known only for 2-chloro- and 2-sulfanylpyridines [14–15], which result in the formation of imidazo[1,2-*a*]pyridines (Scheme 1, reactions 4 and 5). To the best of our knowledge, no successful methods enabling the fusion of a pyrrole or azole ring to a 5-membered heteroaromatic system have been reported to date. The formation of dihydrobenzofuro[3,2-*b*]pyrrole cycloadducts as by-products has been observed in the synthesis of aziridines by the transition metal-catalyzed reaction of benzofurans with 3-arylazirines [16].

This paper presents the use of azirines as annulation reagents for the preparation of 3-aryl-substituted benzo[4,5]thieno[3,2-*b*]pyrroles by the annulation of the 1*H*-pyrrole ring to the benzo[*b*]thiophene system (Scheme 1, reaction 6). The behavior of indoles as aza-analogs of the benzo[*b*]thiophenes under the identified catalytic annulation conditions is also discussed.

## Results and Discussion

Guided by the known fact that 3-arylazirines in the presence of transition metal compounds are sensitive to the enol form of 1,3-dicarbonyls [5,17–19], we chose for our study methyl 3-hydroxybenzo[*b*]thiophene-2-carboxylate (**1**) as an aromatic substrate having a 1,3-dicarbonyl tautomeric form. Initially, it was shown that compound **1** does not react with 3-(*p*-tolyl)-2*H*-azirine (**2a**) when heated at 100 °C in methanol (Table 1, entry 1). No reaction was observed either in the presence of Rh(I), Mn(III), Fe(II), and Cu(II) compounds (Table 1, entries 2‒5). The Cu(I) compounds tested also did not promote the reaction between **1** and **2a**, but some of them were found to catalyze the dimerization of azirine **2a** to 2*H*-imidazole **4** (Table 1, entries 6‒9). Imidazole **4** is a known compound that is formed in low yield upon treatment of azirine **2a** with FeCl_2_ in MeCN [20]. To our delight, the reaction carried out in the presence of 5 mol % of NiSO_4_ gave not only dimer **4**, but also the annulation product, compound **3a**. Further optimization of the reaction conditions aimed at suppressing the formation of dimer **4** showed that nickel chelates exhibit enhanced selectivity in catalyzing the annulation reaction. The highest yield of **3a** (85%) was achieved by treating **1** with 3.2 equiv of the azirine in MeOH at 100 °С with 50 mol % of Ni(hfacac)_2_ (Table 1, entry 13). Lowering the temperature and the catalyst loading resulted in a slight deterioration in the yield and a significant slowing of the reaction, while changing the solvent gave completely unsatisfactory results.

**Table 1:** Optimization of **3a** synthesis ^a^.

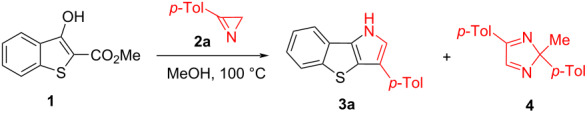					

Entry	Catalyst (mol %)	Azirine (equiv)^b^	Time (h)	Yield of **3a** (%)	Yield of **4** (%)^c^

1	‒	3.2	25	0	0
2	RhCl(PPh_3_)_3_ (5)	1.6	12	0	0
3	Mn(OAc)_3_ (5)	1.6	12	0	0
4	FeCl_2_ (5)	3.2	2	0	0
5	Cu(acac)_2_ (5)	1.6	12	0	0
6	CuBr(PPh_3_)_3_ (5)	3.2	1.5	0	42
7	IPrCuCl (5)	1.6	1.0	0	traces
8	CuI (5)	3.2	2	0	41
9	CuTC (5)	3.2	2	0	45
10	NiSO_4_ (5)	3.2	8	32	12
11	Ni(acac)_2_^d^ (5)	3.2	2	74	16
12	Ni(tfacac)_2_^e^ (5)	3.2	6.5	29	13
13	Ni(hfacac)_2_^f^ (50)	3.2	1.5	85	0
14	Ni(hfacac)_2_ (25)	3.2	2	83	0
15	Ni(hfacac)_2_ (25)	3	3	70	0
16	Ni(hfacac)_2_ (25)	2.5	3	61	0
17	Ni(hfacac)_2_ (50)^g^	3.2	2	0	0

^a^Benzothiophene **1** loading 0.4 mmol. ^b^In portions of 1.6 equiv. ^c^Isolated yields. ^d^acac = acetylacetonate. ^e^tfacac = trifluoroacetylacetonate. ^f^hfacac = hexafluoroacetylacetonate. ^g^Isopropyl alcohol or 1,2-dichloroethane was used as a solvent.

The absence of the methoxycarbonyl group in the obtained product **3a** was unexpected, since there are no examples in the literature of the annulation reactions of cyclic substrates with azirines which are accompanied by the loss of an ester group. In our case, the demethoxycarbonylative annulation enables the formation of aromatic tricyclic benzo[4,5]thieno[3,2-*b*]pyrrole system which is encountered in compounds with antitumor activity [21–22] as well as in compounds exhibiting fluorescent properties [22–23]. Since general methods for the synthesis of benzo[4,5]thieno[3,2-*b*]pyrroles are lacking and obtaining their derivatives remains challenging [24–25], the reaction we discovered could significantly facilitate access to compounds of this type.

**Scheme 2:**
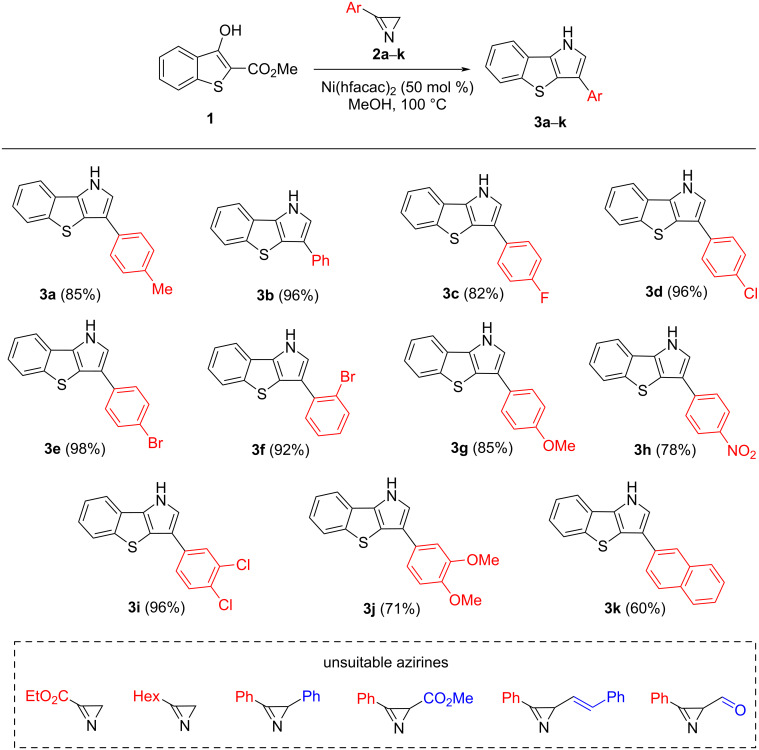
Synthesis of benzo[4,5]thieno[3,2-*b*]pyrroles **3**.

To test the generality and usefulness of the reaction, we investigated a variety of 3-aryl-2*H*-azirines with different substitution patterns of the aryl group (Scheme 2). As the presented data show, the reaction is insensitive to the electronic effects of substituents in the aryl group and, in the majority of cases, gives very high yields of annulation products. The introduction of an *ortho*-substituent into the benzene ring also has no effect on the synthesis efficiency (compound **3f**). A slight decrease in yield was observed only for the naphthyl-substituted annulation product **3k**.

**Scheme 3:**
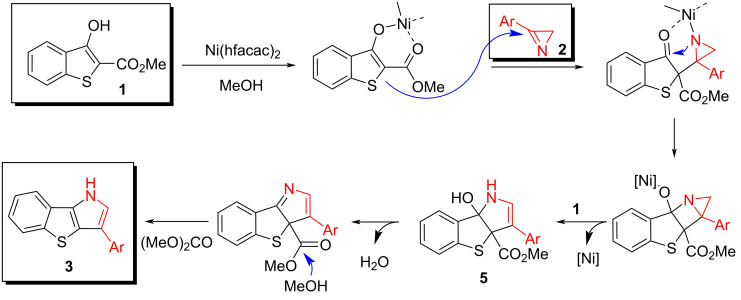
Plausible mechanism for the formation of compounds **3**.

The plausible mechanism of the reaction involves the nucleophilic addition of the Ni-enolate of **1** to the azirine C=N bond, followed by cyclization and the aziridine ring opening into the [3 + 2] cycloaddition product **5** (Scheme 3). It is noteworthy that the annulation proceeds via the azirine N‒C3 bond cleavage. Elimination of the methoxycarbonyl group most likely occurs under the action of methanol. The driving force of this process is the formation of an aromatic thiophene system.

**Scheme 4:**
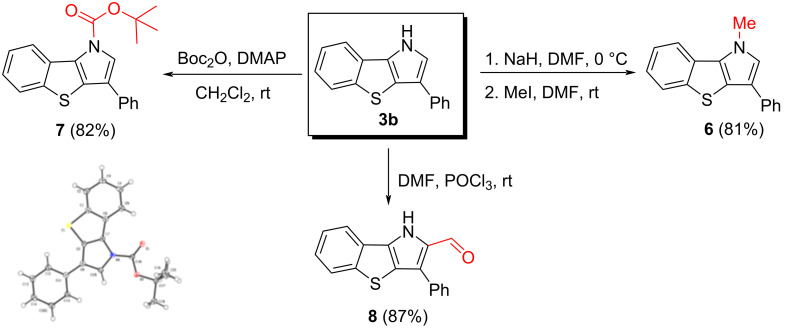
Post-modifications of 1*H*-benzo[4,5]thieno[3,2-*b*]pyrrole (**3b**).

To demonstrate the practicability and synthetic utility of this method for the synthesis of benzo[4,5]thieno[3,2-*b*]pyrrole derivatives, several transformations of compound **3b** were carried out (Scheme 4). Benzothienopyrrole **3b** was methylated using the MeI/NaH system into 1-methyl-substituted derivative **6** in 81% yield. With the same efficiency, when compound **3b** was treated with di-*tert*-butyl dicarbonate in the presence of 4-(dimethylamino)pyridine (DMAP), Boc-derivative **7** was obtained. Finally, the formylation of **3b** under the Vilsmeier–Haack reaction conditions provided aldehyde **8** in high yield.

**Scheme 5:**
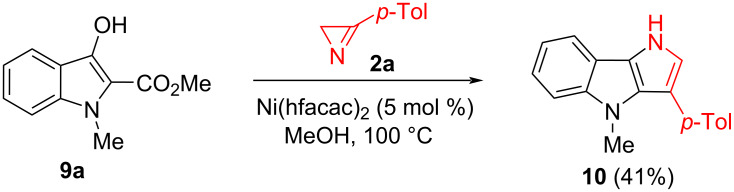
Synthesis of pyrrolo[3,2-*b*]indole **10**.

To further assess the scope and limitations of the developed annulation protocol, we investigated the reactivity of aza-analogs of ester **1**, indoles **9a**‒**c**, toward azirine **2a** under the same conditions. The reaction of *N*-methylindole **9a** with **2a**, carried out in the presence of Ni(hfacac)_2_ (50 mol %), unfortunately, did not give any identifiable products. Experiments with the catalysts presented in Table 1 were also unsuccessful. Nevertheless, favorable outcomes were achieved through a substantial decrease in catalyst loading. Heating of indole **9a** with azirine **2a** in the presence of 5 mol % of Ni(hfacac)_2_ afforded the desired annulation product **10** in 41% yield (Scheme 5).

**Scheme 6:**
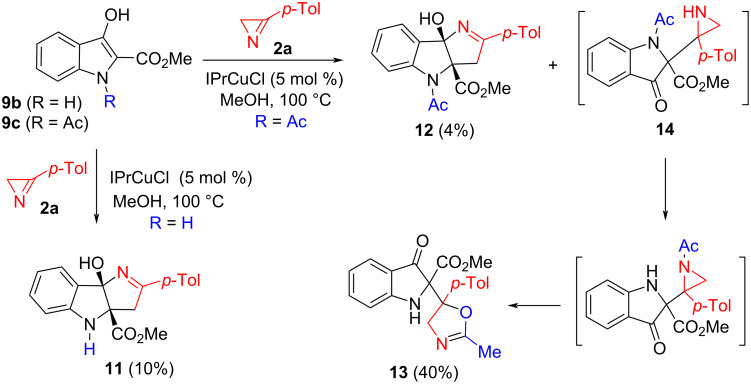
IPrCuCl-catalyzed reactions of indoles **9b**,**c** with azirine **2a**.

Unfortunately, we were unable to find conditions that allowed a similar reaction of NH-indole **9b** to occur. At the same time, when Ni(hfacac)_2_ was replaced with the NHC-complex, IPrCuCl, the formation of cycloadduct **11** was detected, which was isolated in 10% yield (Scheme 6). The formation of this compound implies the cleavage of the azirine N−C2 bond, indicating that the IPrCuCl-catalyzed reaction proceeds by a different mechanism, likely involving the intermediate formation of free radical species [9]. A similar reaction of *N*-acetyl substituted indole **9c** produced [3 + 2] cycloaddition products **12** in even lower yield (4%). However, the main reaction product turned out to be unstable compound **13**, which, nevertheless, was isolated in 40% yield as a single diastereomer and characterized by NMR and HRMS data. Compound **13** is likely formed from aziridine **14** via the N→N acetyl group transfer and subsequent isomerization of the acetylaziridinyl substituent.

**Scheme 7:**
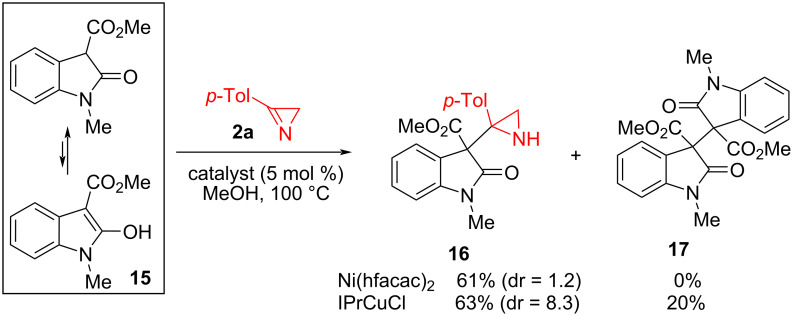
Ni(II)- and Cu(I)-catalyzed reactions of indole **15** with azirine **2a**.

The constitutional isomer of indole **9a**, indole **15**, having a nucleophilic reaction center in the β-position of the indole system, does not give annulation products either with Ni(hfacac)_2_ or with IPrCuCl. In both cases, the main product is aziridine **16** (Scheme 7). It is noteworthy that the IPrCuCl-catalyzed reaction produces aziridine **16** with a high prevalence of one of the diastereomers, while the Ni(hfacac)_2_-catalyzed reaction proceeds with low diastereoselectivity. The significant difference in selectivity can be explained by the fact that, in contrast to the Ni(II)-catalyzed reaction depicted in Scheme 3, the copper(I) catalysis involves the coordination by the metal of both reaction partners, and the formation of the C–C bond between the rings of the reacting molecules occurs in the coordination metal sphere as an intra-, rather than intermolecular, process. The formation of compound **17**, the product of oxidative dimerization of indole-based enol **15**, in the second reaction also points to different mechanisms of the formation of azirindine **16** under Ni(II)- and Cu(I)-catalysis. Oxidative dimerization of non-aromatic cyclic enols has been previously observed in their reactions with 3-arylazirines catalyzed by Cu(I) and Cu(II) complexes and was attributed to the recombination of intermediate free radicals [4].

## Conclusion

In summary, a protocol for the efficient construction of the benzo[4,5]thieno[3,2-*b*]pyrrole skeleton by the annulation reaction of the aromatic system, 3-hydroxybenzo[*b*]thiophene-3-carboxylic ester, with 3-arylazirines has been developed. The annulation is catalyzed by Ni(hfacac)_2_ and proceeds through azirine ring opening across the N–C3 bond. Azirines with both electron-donating and electron-withdrawing C3-aryl substituents tolerate the reaction conditions, and give the annulation products in high yields. The synthesized 3-aryl-1*H*-benzo[4,5]thieno[3,2-*b*]pyrroles can be effectively modified at the positions 1 and 2. The *N*-methylaza analog of the benzo[*b*]thiophene (*N*-methylindole) reacts similarly to provide the annulation product in moderate yield. The described reaction is the first example of a dealkoxycarbonylative annulation reaction using 2*H*-azirines as annulation reagent.

## Supporting Information

Deposition number 2402772 (compound **7**) contains the supplementary crystallographic data for this paper. These data are provided free of charge by the joint Cambridge Crystallographic Data Centre.

File 1 Full experimental details, characterization data and copies of NMR spectra for all new compounds.

## Data Availability

All data that supports the findings of this study is available in the published article and/or the supporting information of this article.
