# Supplementary material for: 3-Aryl-2H-azirines as annulation reagents in the Ni(II)-catalyzed synthesis of 1H-benzo[4,5]thieno[3,2-b]pyrroles
Source: Beilstein J Org Chem. 2025 Aug 11;21:1595–602. doi: 10.3762/bjoc.21.123 (PMC12362299; doi:10.3762/bjoc.21.123)

BEILSTEIN JOURNAL OF ORGANIC CHEMISTRY

Supporting Information

for

3-Aryl-2*H*-azirines as annulation reagents in the Ni(II)-catalyzed synthesis of 1*H*-benzo[4,5]thieno[3,2-*b*]pyrroles

Julia I. Pavlenko, Pavel A. Sakharov, Anastasiya V. Agafonova, Derenik A. Isadzhanyan, Alexander F. Khlebnikov and Mikhail S. Novikov

Beilstein J. Org. Chem. **2025,** *21,* 1595–1602. doi:10.3762/bjoc.21.123

Full experimental details, characterization data and copies of NMR spectra for all new compounds

License and Terms: This is a supporting information file under the terms of the Creative Commons Attribution License (https://creativecommons.org/licenses/by/4.0). Please note that the reuse, redistribution and reproduction in particular requires that the author(s) and source are credited and that individual graphics may be subject to special legal provisions.
The license is subject to the *Beilstein Journal of Organic Chemistry* terms and conditions: (https://www.beilstein-journals.org/bjoc/terms)

Table of contents

1. General experimental details S2
2. Structures of compounds **1**, **2**, **9**, **15** S2
3. Synthesis of thienopyrroles **3** S3
4. Synthesis of thienopyrrole **6** S7
5. Synthesis of thienopyrrole **7** S7
6. Synthesis of thienopyrrole **8** S7
7. Synthesis of pyrroloindole **10** S8
8. Synthesis of pyrroloindole **11** S8
9. Reactions of indole **9c** with azirine **2a** S9
10. Reactions of indole **15** with azirine **2a** S9
11. X-ray data S11
12. References S13
13. ^{1}H and ^{13}C spectra of new compounds S14

1. General experimental details

Melting points were determined on a melting point apparatus and are uncorrected. All chemical shifts (δ) are reported in parts per million (ppm) and the residual solvent peak was used as an internal standard for ^{1}H/^{13}C NMR: $CDCl_3$ δ = 7.28/77.0, DMSO-d_6 δ = 2.50/39.5. High-resolution mass spectra were recorded on an HRMS-ESI-QTOF instrument, electrospray ionization. Thin-layer chromatography (TLC) was conducted on aluminum sheets precoated with SiO_2 ALUGRAM SIL G/UV254. Column chromatography was performed on silica gel 60 M (0.04−0.063 mm). All solvents were distilled and dried prior to use.

Heteroarenols **1**,[1] **9a**,[2] **9b**,[3] **9c**,[4] **15**[5] and azirines **3a–e**,**g**,[6] **3h**,**k**,[7] **3f**,[8] **3i**,[9] **3j**[10] are known compounds and were prepared according to the reported procedures.

2. Structures of compounds 1, 2, 9, 15

Heteroarenols 1, 9a–c, 15

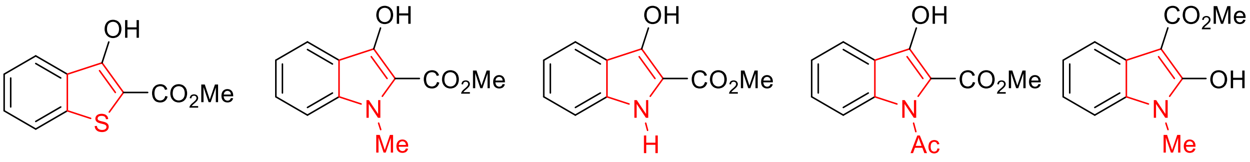

1 **9a** **9b** **9c** **15**

Azirines 2a–k

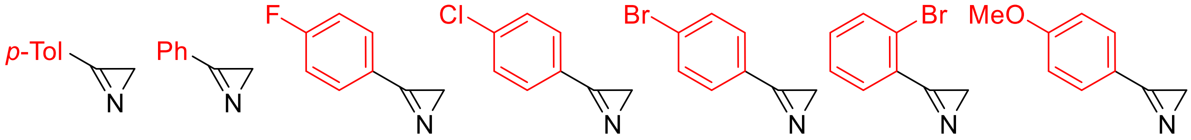

2a **2b** **2c** **2d** **2e** **2f** **2g**

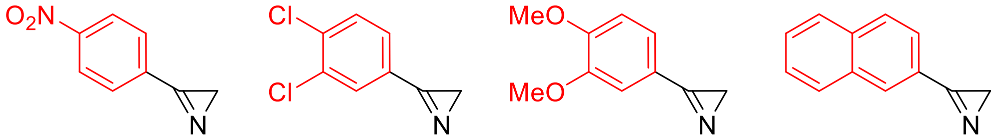

2h **2i** **2j** **2k**

3. Synthesis of thienopyrroles 3

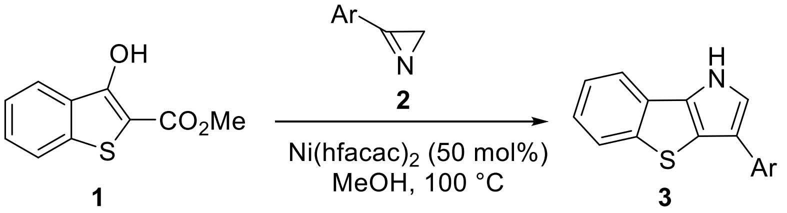

General procedure. A solution of compound **1** (104 mg, 0.5 mmol), azirine **2** (0.8 mmol) and Ni(hfacac)$_2$ (118 mg, 0.25 mmol) in anhydrous MeOH (6 mL) was heated in a screw-cap glass tube at 100 °C for 1 h. Then azirine **2** (0.8 mmol) was added, and the reaction mixture was heated for 0.5 h. After evaporation of the solvent under reduced pressure, the residue was purified by column chromatography on silica gel (eluent PE/EtOAc) and recrystallized from hexane/Et$_2$O to give thienopyrrole **3**.

3-(4-Methylphenyl)-1*H*-benzo[4,5]thieno[3,2-*b*]pyrrole (3a)

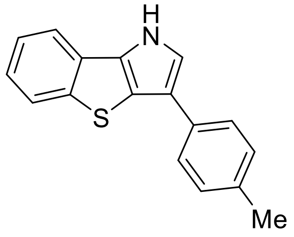

Compound **3a** was obtained as a light green solid (112 mg, 85%) according to the general procedure from **1** and azirine **2a** (210 mg, 1.6 mmol); mp 195–196 °C (hexane/Et$_2$O); ^{1}H NMR (400 MHz, DMSO-d_6) δ 12.09 (s, 1H), 8.02–7.82 (m, 2H), 7.81–7.65 (m, 1H), 7.61–7.49 (m, 2H), 7.47–7.35 (m, 1H), 7.34–7.13 (m, 3H), 2.32 (s, 3H); ^{13}C{^{1}H} NMR (101 MHz, DMSO-d_6) 140.7, 134.4, 132.8, 131.6, 129.4, 127.0, 124.4, 124.2, 124.0, 122.7, 120.4, 118.6, 118.1, 116.9, 20.7; HRMS−ESI [M – H]$^-$ calcd for $C_{17}H_{12}NS^-$ 262.0696, found 262.0689.

3-Phenyl-1*H*-benzo[4,5]thieno[3,2-*b*]pyrrole (3b)

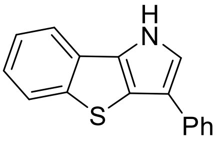

Compound **3b** was obtained as a gray solid (120 mg, 96%) according to the general procedure from **1** and azirine **2b** (187 mg, 1.6 mmol); mp 183–184 °C (hexane/Et$_2$O); ^{1}H NMR (400 MHz, DMSO-d_6) δ 12.16 (s, 1H), 8.07–7.86 (m, 2H), 7.78 (s, 1H), 7.73–7.57 (m, 2H), 7.55–7.33 (m, 3H), 7.33–7.12 (m, 2H); ^{13}C{^{1}H} NMR (101 MHz, DMSO-d_6) δ 140.7, 134.4, 133.0, 128.9, 126.9, 125.3, 124.4, 124.2, 124.1, 122.8, 120.9, 118.7, 118.1, 116.8; HRMS−ESI [M + H]$^+$ calcd for $C_{16}H_{12}NS^+$ 250.0685, found 250.0680.

3-(4-Fluorophenyl)-1*H*-benzo[4,5]thieno[3,2-*b*]pyrrole (3c)

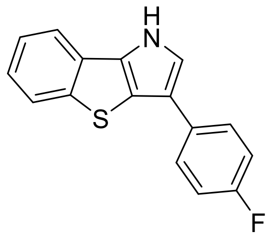

Compound **3c** was obtained as a gray solid (109 mg, 82%) according to the general procedure from **1** and azirine **2c** (216 mg, 1.6 mmol); mp 207–208 °C (hexane/Et_2O); ^{1}H NMR (400 MHz, DMSO-*d*$_6$) δ 12.14 (s, 1H), 7.98–7.84 (m, 2H), 7.80–7.71 (m, 1H), 7.69–7.60 (m, 2H), 7.47–7.35 (m, 1H), 7.34–7.21 (m, 3H); ^{13}C{^{1}H} NMR (101 MHz, DMSO-*d*$_6$) δ 160.2 (d, *J* = 242.3 Hz), 140.6, 133.0, 131.0 (d, *J* = 3.5 Hz), 126.9, 125.9 (d, *J* = 7.7 Hz), 124.5, 124.1, 122.8, 120.8, 118.7, 118.0, 115.9, 115.7 (d, *J* = 21.5 Hz); HRMS–ESI $[M – H]^–$ calcd for $C_{16}H_9FNS^–$ 266.0445, found 266.0450.

3-(4-Chlorophenyl)-1*H*-benzo[4,5]thieno[3,2-*b*]pyrrole (3d)

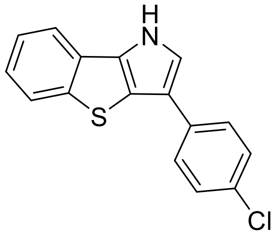

Compound **3d** was obtained as a brown solid (136 mg, 96%) according to the general procedure from **1** and azirine **2d** (242 mg, 1.6 mmol); mp 232–234 °C (hexane/Et_2O); ^{1}H NMR (400 MHz, DMSO-*d*$_6$) δ 12.23 (s, 1H), 7.99–7.88 (m, 2H), 7.86–7.78 (m, 1H), 7.71–7.59 (m, 2H), 7.55–7.46 (m, 2H), 7.46–7.39 (m, 1H), 7.33–7.22 (m, 1H); ^{13}C{^{1}H} NMR (101 MHz, DMSO-*d*$_6$) δ 140.7, 133.4, 133.1, 129.5, 128.9, 126.8, 125.8, 124.5, 124.1, 122.9, 121.4, 118.7, 118.1, 115.7; HRMS–ESI $[M + H]^+$ calcd for $C_{16}H_{11}{}^{35}ClNS^+$ 284.0295, found 284.0296.

3-(4-Bromophenyl)-1*H*-benzo[4,5]thieno[3,2-*b*]pyrrole (3e)

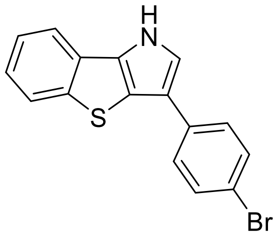

Compound **3e** was obtained as a gray solid (161 mg, 98%) according to the general procedure from **1** and azirine **2e** (314 mg, 1.6 mmol); mp 233–235 °C (hexane/Et_2O); ^{1}H NMR (400 MHz, DMSO-*d*$_6$) δ 12.26 (s, 1H), 7.98–7.87 (m, 2H), 7.83 (s, 1H), 7.67–7.53 (m, 4H), 7.47–7.38 (m, 1H), 7.33–7.23 (m, 1H); ^{13}C{^{1}H} NMR (101 MHz, DMSO-*d*$_6$) δ 140.7, 133.8, 133.1, 131.8, 126.8, 126.1, 124.5, 124.1, 122.9, 121.5, 118.7, 118.0, 117.8, 115.7; HRMS–ESI $[M – H]^–$ calcd for $C_{16}H_9{}^{81}BrNS^–$ 327.9624, found 327.9622.

3-(2-Bromophenyl)-1*H*-benzo[4,5]thieno[3,2-*b*]pyrrole (3f)

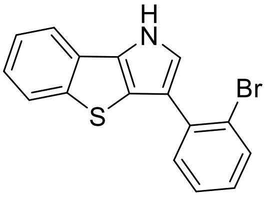

Compound **3f** was obtained as a gray solid (151 mg, 92%) according to the general procedure from **1** and azirine **2f** (314 mg, 1.6 mmol); mp 151–152 °C (hexane/Et_2O); ^{1}H NMR (400 MHz, DMSO-d_6) δ 12.22 (s, 1H), 7.98–7.83 (m, 2H), 7.78–7.69 (m, 1H), 7.65–7.56 (m, 2H), 7.51–7.36 (m, 2H), 7.31–7.19 (m, 2H); ^{13}C{^{1}H} NMR (101 MHz, DMSO-d_6) δ 140.7, 135.2, 133.6, 132.1, 130.4, 128.03, 127.96, 127.0, 124.3, 123.9, 123.5, 122.8, 121.2, 120.6, 118.7, 116.0; HRMS−ESI [M + H]$^+$ calcd for $C_{16}H_{11}{}^{81}BrNS^+$ 329.9770, found 329.9774.

3-(4-Methoxyphenyl)-1*H*-benzo[4,5]thieno[3,2-*b*]pyrrole (3g)

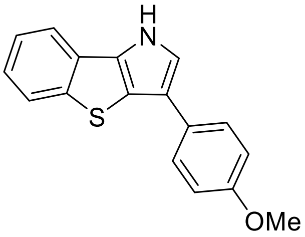

Compound **3g** was obtained as a gray solid (119 mg, 85%) according to the general procedure from **1** and azirine **2g** (235 mg, 1.6 mmol); mp 203–204 °C (hexane/Et_2O); ^{1}H NMR (400 MHz, DMSO-d_6) δ 12.03 (s, 1H), 7.99–7.82 (m, 2H), 7.71–7.60 (m, 1H), 7.60–7.50 (m, 2H), 7.47–7.35 (m, 1H), 7.32–7.22 (m, 1H), 7.09–6.95 (m, 2H), 3.78 (s, 3H); ^{13}C{^{1}H} NMR (101 MHz, DMSO-d_6) δ 157.2, 140.7, 132.8, 127.1, 127.0, 125.5, 124.4, 124.1, 122.7, 119.9, 118.6, 117.9, 116.7, 114.4, 55.1; HRMS−ESI [M + H]$^+$ calcd for $C_{17}H_{14}NOS^+$ 280.0791, found 280.0787.

3-(4-Nitrophenyl)-1*H*-benzo[4,5]thieno[3,2-*b*]pyrrole (3h)

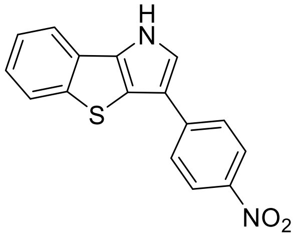

Compound **3h** was obtained as a brown solid (115 mg, 78%) according to the general procedure from **1** and azirine **2h** (259 mg, 1.6 mmol); mp 221–222 °C (hexane/Et_2O); ^{1}H NMR (400 MHz, DMSO-d_6) δ 12.53 (s, 1H), 8.34–8.26 (m, 2H), 8.10–8.03 (m, 1H), 8.01–7.90 (m, 2H), 7.89–7.74 (m, 2H), 7.52–7.38 (m, 1H), 7.38–7.24 (m, 1H); ^{13}C{^{1}H} NMR (101 MHz, DMSO-d_6) δ 144.2, 141.5, 140.7, 133.6, 126.6, 124.7, 124.5, 124.4, 124.1, 124.0, 123.2, 118.9, 118.6, 115.2; HRMS−ESI [M – H]$^-$ calcd for $C_{16}H_9N_2O_2S^-$ 293.0390, found 293.0389.

3-(3,4-Dichlorophenyl)-1*H*-benzo[4,5]thieno[3,2-*b*]pyrrole (3i)

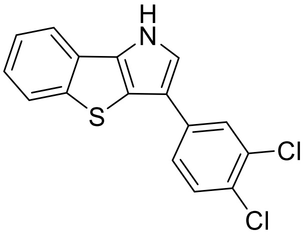

Compound **3i** was obtained as a brown solid (153 mg, 96%) according to the general procedure from **1** and azirine **2i** (298 mg, 1.6 mmol); mp 226–228 °C (hexane/Et_2O); ^{1}H NMR (400 MHz, DMSO-d_6) δ 12.34 (s, 1H), 8.01–7.88 (m, 3H), 7.88–7.83 (m, 1H), 7.74–7.65 (m, 1H), 7.62–7.53 (m, 1H), 7.48–7.38 (m, 1H), 7.34–7.24 (m, 1H); ^{13}C{^{1}H} NMR (101 MHz, DMSO-d_6) δ 140.6, 135.4, 133.2, 131.7, 131.1, 127.1, 126.7, 125.6, 124.6, 124.2, 124.1, 123.0, 122.4, 118.8, 118.1, 114.5; HRMS−ESI [M + H]$^+$ calcd for $C_{16}H_{10}{}^{35}Cl_2NS^+$ 317.9906, found 317.9901.

3-(3,4-Dimethoxyphenyl)-1*H*-benzo[4,5]thieno[3,2-*b*]pyrrole (3j)

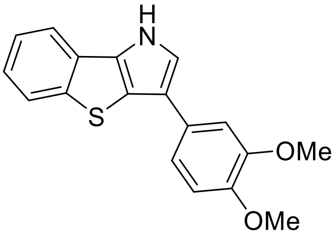

Compound **3j** was obtained as a gray solid (110 mg, 71%) according to the general procedure from **1** and azirine **2j** (283 mg, 1.6 mmol); mp 181–183 °C (hexane/Et_2O); ^{1}H NMR (400 MHz, DMSO-d_6) δ 12.05 (s, 1H), 8.00–7.82 (m, 2H), 7.77–7.63 (m, 1H), 7.49–7.33 (m, 1H), 7.32–7.18 (m, 2H), 7.17–7.07 (m, 1H), 7.07–6.98 (m, 1H), 3.86 (s, 3H), 3.78 (s, 3H); ^{13}C{^{1}H} NMR (101 MHz, DMSO-d_6) δ 149.2, 146.9, 140.7, 132.8, 127.6, 127.0, 124.4, 124.1, 122.7, 120.2, 118.6, 118.1, 117.0, 116.5, 112.5, 108.5, 55.6, 55.5; HRMS−ESI [M + H]$^+$ calcd for $C_{18}H_{16}NO_2S^+$ 310.0896, found 310.0899.

3-(Naphthalen-2-yl)-1*H*-benzo[4,5]thieno[3,2-*b*]pyrrole (3k)

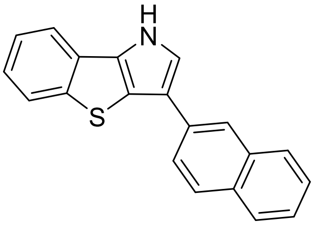

Compound **3k** was obtained as a gray solid (90 mg, 60%) according to the general procedure from **1** and azirine **2k** (267 mg, 1.6 mmol); mp 241–243 °C (hexane/Et_2O); ^{1}H NMR (400 MHz, DMSO-d_6) δ 12.24 (s, 1H), 8.04–7.87 (m, 8H), 7.56–7.49 (m, 1H), 7.49–7.40 (m, 2H), 7.35–7.25 (m, 1H); ^{13}C{^{1}H} NMR (101 MHz, DMSO-d_6) δ 140.8, 133.6, 133.2, 132.1, 131.3, 128.4, 127.6, 127.4, 126.9, 126.5, 125.1, 124.5, 124.1, 123.7, 122.9, 121.7, 121.5, 118.7, 118.3, 116.8; HRMS−ESI [M + H]$^+$ calcd for $C_{20}H_{14}NS^+$ 300.0841, found 300.0843.

4. Synthesis of 1-methyl-3-phenyl-1*H*-benzo[4,5]thieno[3,2-*b*]pyrrole (6)

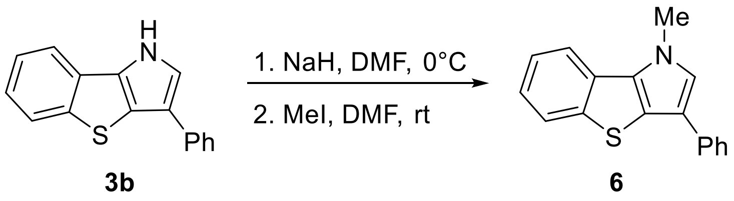

To a solution of compound **3b** (50 mg, 0.2 mmol) in DMF (2 mL) cooled to 0 °C, NaH (60% in mineral oil, 16 mg, 0.4 mmol) was added and the reaction mixture was stirred for 20 min at the same temperature. Methyl iodide (48 mg, 0.34 mmol) was added and the reaction mixture was stirred at room temperature for 1 h. After the reaction completion, the reaction mixture was washed with saturated NH_4Cl solution (10 mL) and extracted with EtOAc (3 × 10 mL). The combined organic layer was washed with brine, dried over Na_2SO_4 and concentrated under reduced pressure. The residue was purified by column chromatography on silica gel (eluent PE/EtOAc) to give compound **6** (43 mg, 81%) as a white solid; mp 131–133 °C (EtOAc); ^{1}H NMR (400 MHz, DMSO-d_6) δ 8.09–8.00 (m, 1H), 8.00–7.92 (m, 1H), 7.71 (s, 1H), 7.65–7.54 (m, 2H), 7.49–7.38 (m, 3H), 7.34–7.27 (m, 1H), 7.24–7.17 (m, 1H), 4.12 (s, 3H); ^{13}C{^{1}H} NMR (101 MHz, DMSO-d_6) δ 140.8, 134.1, 132.9, 129.0, 126.9, 125.8, 125.4, 124.5, 124.2, 124.1, 122.8, 118.9, 118.4, 115.7, 35.6; HRMS−ESI $[M + H]^+$ calcd for $C_{17}H_{14}NS^+$ 264.0841, found 264.0841.

5. Synthesis of *tert*-butyl 3-phenyl-1*H*-benzo[4,5]thieno[3,2-*b*]pyrrole-1-carboxylate (7)

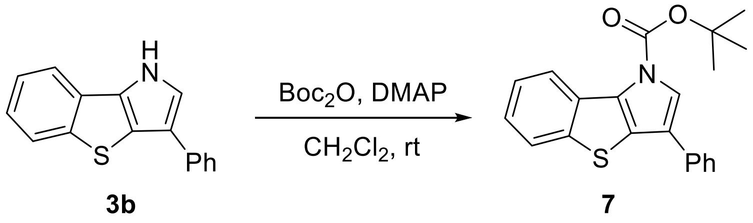

To a solution of **3b** (50 mg, 0.2 mmol) and dimethylaminopyridine (2 mg, 0.02 mmol) in CH_2Cl_2 (5 mL), di-*tert*-butyl dicarbonate (65 mg, 0.3 mmol) was added, and the reaction mixture was stirred for 1.5 h. Then, the reaction mixture was washed with saturated NH_4Cl solution (10 mL), and the aqueous layer was extracted with CH_2Cl_2 (3 × 10 mL). The combined organic layer was washed with brine, dried over Na_2SO_4 and concentrated under reduced pressure. The residue was purified by column chromatography on silica gel (eluent PE/EtOAc) to give compound **7** (57 mg, 82%) as a yellow solid; mp 161–163 °C (EtOAc); ^{1}H NMR (400 MHz, DMSO-d_6) δ 8.84–8.65 (m, 1H), 8.07 (s, 1H), 8.04–7.97 (m, 1H), 7.84–7.69 (m, 2H), 7.56–7.43 (m, 3H), 7.43–7.30 (m, 2H), 1.69 (s, 9H); ^{13}C{^{1}H} NMR (101 MHz, DMSO-d_6) δ 148.3, 141.3, 132.0, 131.6, 129.1, 127.3, 126.9, 125.3, 124.7, 124.6, 124.0, 123.9, 123.1, 122.5, 120.0, 85.0, 27.6; HRMS−ESI $[M + H]^+$ calcd for $C_{21}H_{20}NO_2S^+$ 350.1209, found 350.1200.

6. Synthesis of 3-phenyl-1*H*-benzo[4,5]thieno[3,2-*b*]pyrrole-2-carbaldehyde (8)

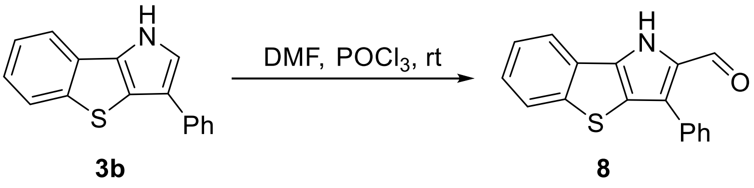

To a solution of **3b** (50 mg, 0.2 mmol) in DMF (2 mL), $POCl_3$ (61 mg, 0.4 mmol) was added, and the reaction mixture was stirred for 12 h. Then, the reaction mixture was washed with saturated $NaHCO_3$ solution (10 mL) and extracted with EtOAc (3 × 10 mL). The combined organic layer was washed with brine, dried over Na_2SO_4 and concentrated under reduced pressure. The residue was purified by column chromatography on silica gel (eluent PE/EtOAc) to give compound **8** (48 mg, 87%) as a brown solid; mp 277–279 °C (EtOAc); ^{1}H NMR (400 MHz, DMSO-d_6) δ 13.16 (s, 1H), 9.75 (s, 1H), 8.30–8.13 (m, 1H), 8.03–7.93 (m, 1H), 7.78–7.65 (m, 2H), 7.62–7.52 (m, 2H), 7.52–7.38 (m, 3H); ^{13}C{^{1}H} NMR (101 MHz, DMSO-d_6) δ 179.7, 143.3, 137.6, 132.2, 132.0, 129.2, 128.8, 128.0, 127.3, 126.0, 125.8, 125.0, 124.4, 121.9, 121.5; HRMS−ESI [M + H]$^+$ calcd for $C_{17}H_{12}NOS^+$ 278.0634, found 278.0630.

7. Synthesis of 4-methyl-3-(4-methylphenyl)-1,4-dihydropyrrolo[3,2-*b*]indole (10)

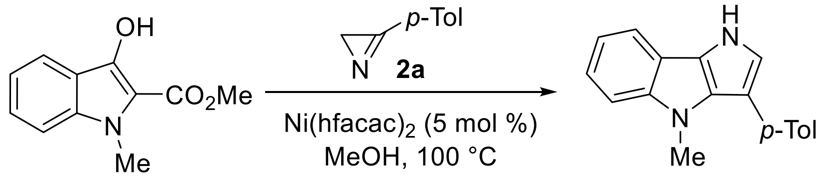

9a **10**

A solution of enol **9a** (41 mg, 0.2 mmol), azirine **2a** (42 mg, 0.32 mmol) and Ni(hfacac)$_2$ (5 mg, 0.01 mmol) in anhydrous MeOH (3 mL) was heated in a screw-cap glass tube at 100 °C for 20 min. After evaporation of the solvent under reduced pressure, the residue was purified by column chromatography on silica gel (eluent PE/EtOAc) and recrystallized from hexane/Et_2O to give compound **10** as a colorless solid (21 mg, 41%); mp 169–170 °C (hexane/Et_2O); ^{1}H NMR (400 MHz, $CDCl_3$) δ 8.10 (s, 1H), 7.70–7.63 (m, 1H), 7.62–7.52 (m, 2H), 7.46–7.40 (m, 1H), 7.38–7.30 (m, 3H), 7.25–7.17 (m, 1H), 7.00–6.87 (m, 1H), 3.86 (s, 3H), 2.52 (s, 3H); ^{13}C{^{1}H} NMR (101 MHz, $CDCl_3$) δ 141.9, 135.6, 132.3, 131.9, 129.2, 128.7, 120.7, 120.5, 119.9, 118.0, 116.4, 115.2, 110.3, 109.3, 31.7, 21.1; HRMS-ESI [M + H]$^+$ calcd for $C_{18}H_{17}N_2^+$ 261.1386, found 261.1391.

8. Synthesis of methyl *rac*-(3a*R*,8b*S*)-8b-hydroxy-2-(4-methylphenyl)-4,8b-dihydropyrrolo[3,2-*b*]indole-3a(3*H*)-carboxylate (11)

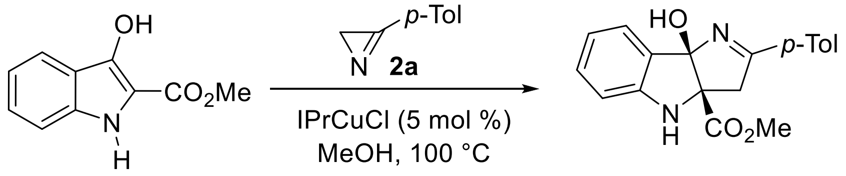

9b **11**

A solution of enol **9b** (38 mg, 0.2 mmol), azirine **2a** (42 mg, 0.32 mmol) and IPrCuCl (4.9 mg, 0.01 mmol) in anhydrous MeOH (3 mL) was heated in a screw-cap glass tube at 100 °C for 20 min. After evaporation of the solvent under reduced pressure, the residue was purified by column chromatography on silica gel (eluent PE/EtOAc) to give compound **11** as a light yellow oil (5 mg, 10%); ^{1}H NMR (400 MHz, $CDCl_3$) δ 7.77–7.68 (m, 2H), 7.37–7.31 (m, 1H), 7.26–7.19 (m, 3H), 6.91–6.84 (m, 1H), 6.82–6.76 (m, 1H) 5.19 (br s, 1H), 3.85 (s, 3H), 3.73 and 3.67 (AB-q, J = 17.0 Hz, 2H), 2.39 (s, 3H); ^{13}C{^{1}H} NMR (101 MHz, $CDCl_3$) δ 175.7, 170.0, 148.1, 142.1, 130.9, 130.7, 130.5, 129.2, 128.0, 124.4, 120.6, 112.0, 100.8, 89.5, 53.1, 48.4, 21.5; HRMS-ESI [M + H]$^+$ calcd for $C_{19}H_{19}N_2O_3^+$ 323.1390, found 323.1403.

9. Reactions of indole 9c with azirine 2a

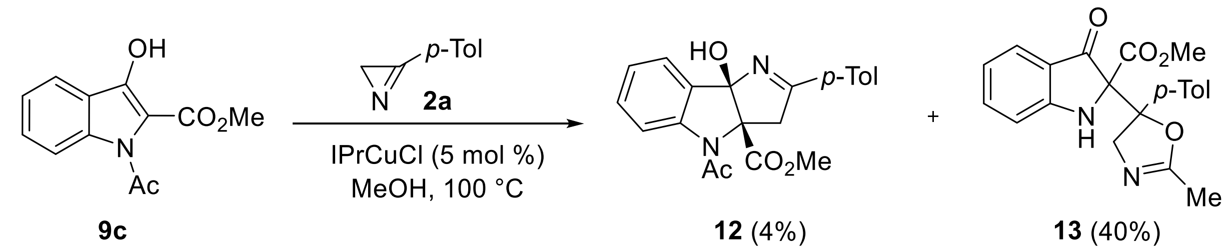

A solution of compound **9c** (47 mg, 0.2 mmol), azirine **2a** (42 mg, 0.32 mmol) and IPrCuCl (4.9 mg, 0.01 mmol) in anhydrous MeOH (3 mL) was heated in a screw-cap glass tube at 100 °C for 20 min. After evaporation of the solvent under reduced pressure, the residue was purified by column chromatography on silica gel (eluent PE/EtOAc) to give pyrroloindole **12** as a yellow oil (3 mg, 4%) and oxazole **13** as a colorless solid (29 mg, 40%).

Methyl (3a*R*,8b*S*)-4-acetyl-8b-hydroxy-2-(4-methylphenyl)-4,8b-dihydropyrrolo[3,2-*b*]indole-3a(3*H*)-carboxylate (12). Yellow oil. ^{1}H NMR (400 MHz, $CDCl_3$) δ 7.86–7.80 (m, 1H), 7.74–7.68 (m, 2H), 7.44–7.37 (m, 1H), 7.25–7.19 (m, 4H), 4.60 (d, J = 19.1 Hz, 1H), 3.77 (s, 3H), 3.44 (d, J = 19.1 Hz, 1H), 2.55 (s, 3H), 2.40 (s, 3H); ^{13}C{^{1}H} NMR (101 MHz, $CDCl_3$) δ 173.5, 169.1, 168.4, 142.6, 140.8, 132.1, 130.7, 129.6, 129.3, 128.4, 126.2, 124.2, 113.4, 109.9, 78.4, 53.0, 47.6, 25.7, 21.6; HRMS-ESI [M + H]$^+$ calcd for $C_{21}H_{21}N_2O_4^+$ 365.1496, found 365.1479.

Methyl 2-(2-methyl-5-(4-methylphenyl)-4,5-dihydrooxazol-5-yl)-3-oxoindoline-2-carboxylate (13). Colorless solid, decomposes slowly at room temperature. ^{1}H NMR (400 MHz, $CDCl_3$) δ 7.61–7.53 (m, 1H), 7.52–7.44 (m, 1H), 7.34–7.28 (m, 2H), 7.23–7.12 (m, 2H), 6.95–6.83 (m, 2H), 5.48 (d, J = 14.5 Hz, 1H), 4.97 (s, 1H), 4.17 (d, J = 14.5 Hz, 1H), 3.75 (s, 3H), 2.36 (s, 3H), 1.87 (s, 3H); ^{13}C{^{1}H} NMR (101 MHz, $CDCl_3$) δ 192.0, 166.2, 162.2, 161.0, 138.3, 138.1, 137.5, 129.0, 126.0, 125.1, 120.5, 120.0, 112.9, 90.5, 76.4, 62.5, 53.4, 21.0, 13.9; HRMS-ESI [M + Na]$^+$ calcd for $C_{21}H_{20}N_2NaO_4^+$ 387.1315, found 387.1321.

10. Reactions of indole 15 with azirine 2a

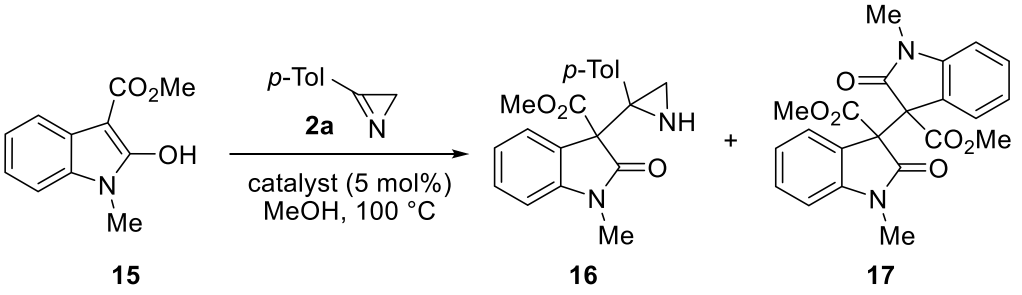

	16	17
IPrCuCl	63% (*dr* = 8.3)	20%
$Ni(hfacac)_2$	61% (*dr* = 1.2)	0%

A solution of compound **15** (41 mg, 0.2 mmol), azirine **2a** (42 mg, 0.32 mmol) and IPrCuCl (4.9 mg, 0.01 mmol) in anhydrous MeOH (3 mL) was heated in a screw-cap glass tube at 100 °C for 10 min. After evaporation of the solvent under reduced pressure, the residue was purified by column chromatography on silica gel (eluent PE/EtOAc) to give aziridine **16** as a yellow oil (dr = 8.3, 42 mg, 63%) and biindoline **17**[5] as a colorless solid (8 mg, 20%). The reaction of **15** with azirine **2a** catalyzed by $Ni(hfacac)_2$ (5 mg, 0.01 mmol) gave aziridine **16** (dr = 1.2, 41 mg, 61%).

Methyl 1-methyl-3-[2-(4-methylphenyl)aziridin-2-yl]-2-oxoindoline-3-carboxylate (16). Signals of major isomer: ^{1}H NMR (400 MHz, $CDCl_3$) δ 7.35–7.28 (m, 1H), 7.16–7.05 (m, 2H),

7.03–6.89 (m, 4H), 6.78–6.69 (m, 1H), 3.69 (s, 3H), 3.09 (s, 3H), 2.67 (s, 1H), 2.29 (s, 3H), 1.93 (s, 1H), 1.54 (br s, 1H); $^{13}C\{^1H\}$ NMR (101 MHz, $CDCl_3$) δ 171.1, 167.9, 144.0, 137.7, 136.2, 129.9, 129.1, 128.1, 126.5, 126.0, 121.9, 107.9, 62.6, 52.8, 43.9, 28.3, 26.3, 21.0; HRMS-ESI $[M + Na]^+$ calcd for $C_{20}H_{20}N_2NaO_3^+$ 359.1366, found 359.1368.

11. X-ray data

Compound 7 (CCDC 2402772)

Single crystals of **7** were grown by slow evaporation of its solution in Et_2O-hexane mixture A suitable crystal was selected and intensity data were collected on a XtaLAB Synergy, Single source at home/near, HyPix diffractometer. The crystal was kept at 99.98(10) K during data collection. Using Olex2 [12], the structure was solved with the SHELXT [13] structure solution program using Intrinsic Phasing and refined with the olex2.refine [14] refinement package using Gauss-Newton minimization.

Figure S-1. X-Ray crystal structure of compound **7** with 50% ellipsoid probability (CCDC 2402772)

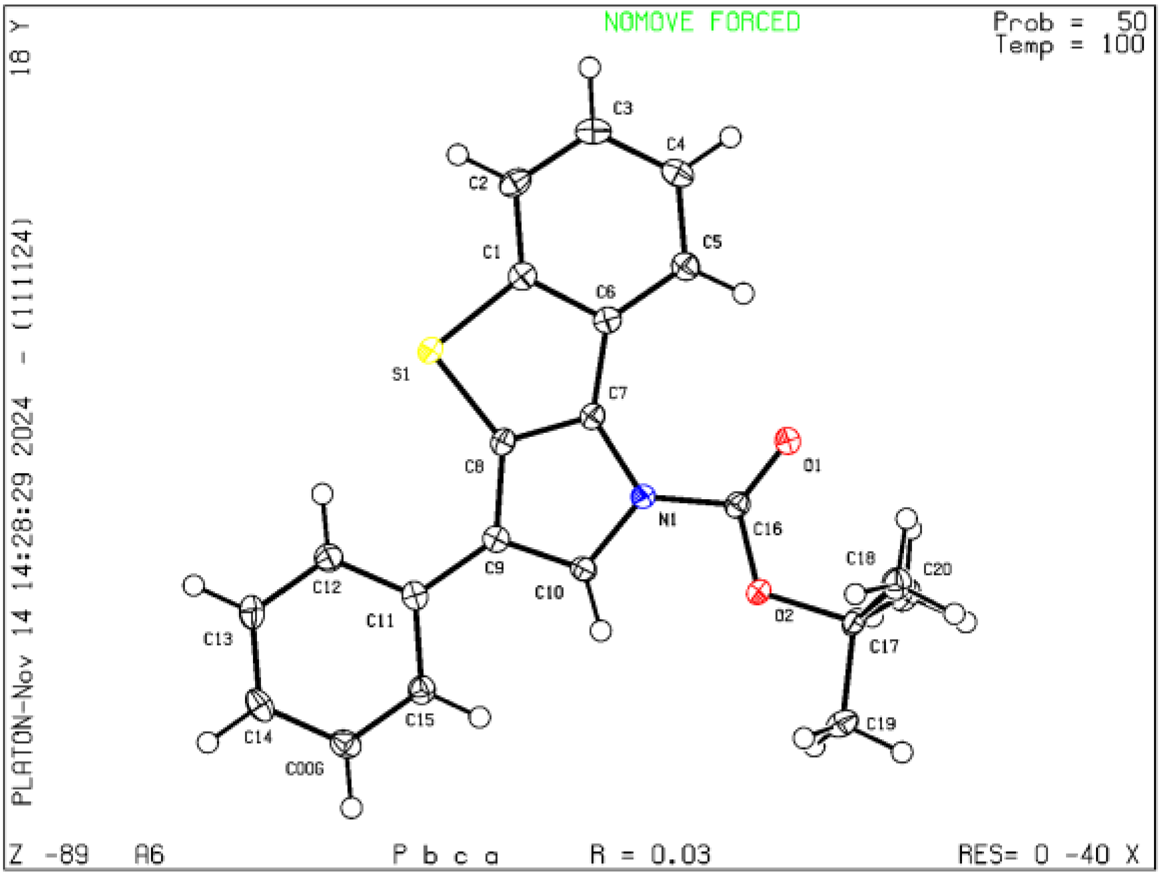

Table S-1. Crystal data and structure refinement for **7**

Empirical formula	$C_{21}H_{19}NO_2S$
Formula weight	349.456
Temperature/K	99.98(10)
Crystal system	orthorhombic
Space group	Pbca
a/Å	9.7785(1)
b/Å	13.9878(1)
c/Å	25.3803(2)
α/°	90
β/°	90
γ/°	90

Volume/Å^3	3471.51(5)
Z	8
ρ_{calc}g/cm^3	1.337
μ/mm^{-1}	1.764
F(000)	1479.1
Crystal size/mm^3	0.16 × 0.14 × 0.12
Radiation	Cu Kα (λ = 1.54184)
2Θ range for data collection/°	6.96 to 144.92
Index ranges	$-7 \le h \le 12$, $-16 \le k \le 17$, $-31 \le l \le 31$
Reflections collected	20991
Independent reflections	3424 [R_{int} = 0.0473, R_{sigma} = 0.0251]
Data/restraints/parameters	3424/0/229
Goodness-of-fit on F^2	1.043
Final R indexes [I>=2σ (I)]	R_1 = 0.0315, wR_2 = 0.0811
Final R indexes [all data]	R_1 = 0.0330, wR_2 = 0.0824
Largest diff. peak/hole / e Å^{-3}	0.26/-0.31

12. References

[1] P. Gopinath, S. Nilaya, T. R. Debi, V. Ramkumar, K. M. Muraleedharan "As Many as Six Tandem Reactions in One Step! Unprecedented Formation of Highly Functionalized Benzothiophenes" *Chem. Commun.* **2009**, *46*, 7131–7133.

[2] H. Kang, A. L.Jemison, E. Nigro, M. C. Kozlowski "Oxidative Coupling of 3-Oxindoles with Indoles and Arenes" *ChemSusChem* **2019**, *12*, 3144–3151.

[3] M. Jessing, P. S. Baran "Oxidative Coupling of Indoles with 3-Oxindoles" *Heterocycles* **2010**, *82*, 1739–1745.

[4] S. Yarlagadda, B. Ramesh, C. Ravikumar Reddy, L. Srinivas, B. Sridhar, B. V. Subba Reddy "Organocatalytic Enantioselective Amination of 2-Substituted Indolin-3-Ones: A Strategy for the Synthesis of Chiral α-Hydrazino Esters" *Org. Lett.* **2017**, *19*, 170–173.

[5] S. Ghosh, S. Chaudhuri, A. Bisai "Oxidative Dimerization of 2-Oxindoles Promoted by KO^{t}Bu-I_2: Tot al Synthesis of (±)-Folicanthine" *Org. Lett.* **2015**, *17*, 1373–1376.

[6] A. G. Hortmann, D. A.Robertson, B. K. Gillard "A Convenient Procedure for the Preparation of 2-Arylazirines" *J. Org. Chem* **1979**, *87*, 322–324.

[7] Y. Wang, X. Lei, Y. Tang "Rh(II)-Catalyzed Cycloadditions of 1-Tosyl 1,2,3-Triazoles with 2*H*-Azirines: Switchable Reactivity of Rh-Azavinylcarbene as [2C]- or Aza-[3C]-Synthon" *Chem. Commun.* **2015**, *51*, 4507–4510.

[8] A. F. Khlebnikov, M. S. Novikov, P. P. Petrovskii, H. Stoeckli-Evans "An Aza Cyclopropylcarbinyl-Homoallyl Radical Rearrangement-Radical Cyclization Cascade. Synthesis of Dibenzoimidazoazepine and Oxazepine Derivatives" *J. Org. Chem.* **2011**, *76*, 5384–5391.

[9] A. F. Khlebnikov, M. S. Novikov, A. A. Amer "Generation and Cycloadditions of Azirinium Difluoromethanides-Strained Azomethine Ylides" *Tetrahedron Lett.* **2002**, *43*, 8523–8525.

[10] P. A.Sakharov, N. V. Rostovskii, A. F. Khlebnikov, O. V. Khoroshilova, M. S. Novikov "Transition Metal-Catalyzed Synthesis of 3-Coumaranone-Containing NH-Aziridines from 2*H*-Azirines: Nickel(II) versus Gold(I)" *Adv Synth. Catal.* **2019**, *361*, 3359–3372.

[12] O. V. Dolomanov, L. J. Bourhis, R. J. Gildea, J. A. K. Howard, H. Puschmann "OLEX2: a Complete Structure Solution, Refinement and Analysis Program" *J. Appl. Cryst.* **2009**, *42*, 339–341.

[13] G. M. Sheldrick "SHELXT – Integrated Space-Group and Crystal-Structure Determination" *Acta Cryst.* **2015**, *A71*, 3–8.

[14] G. M. Sheldrick "Crystal Structure Refinement with SHELXL" *Acta Cryst.* **2015**, *C71*, 3–8.

13. ¹H and ¹³C spectra of new compounds

^{1}H NMR spectrum of **3a** (400 MHz, DMSO-d_6)

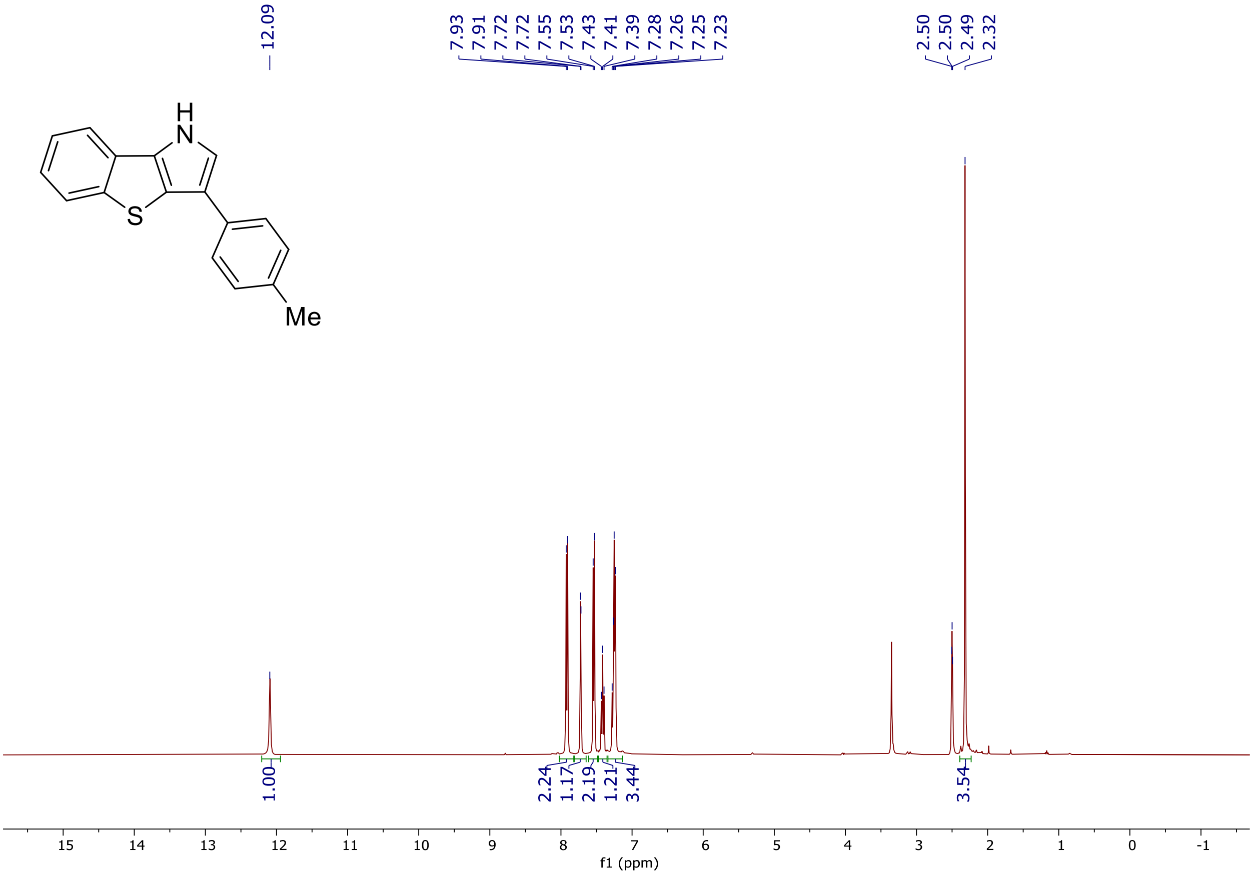

^{13}C{^{1}H} NMR spectrum of **3a** (101 MHz, DMSO-d_6)

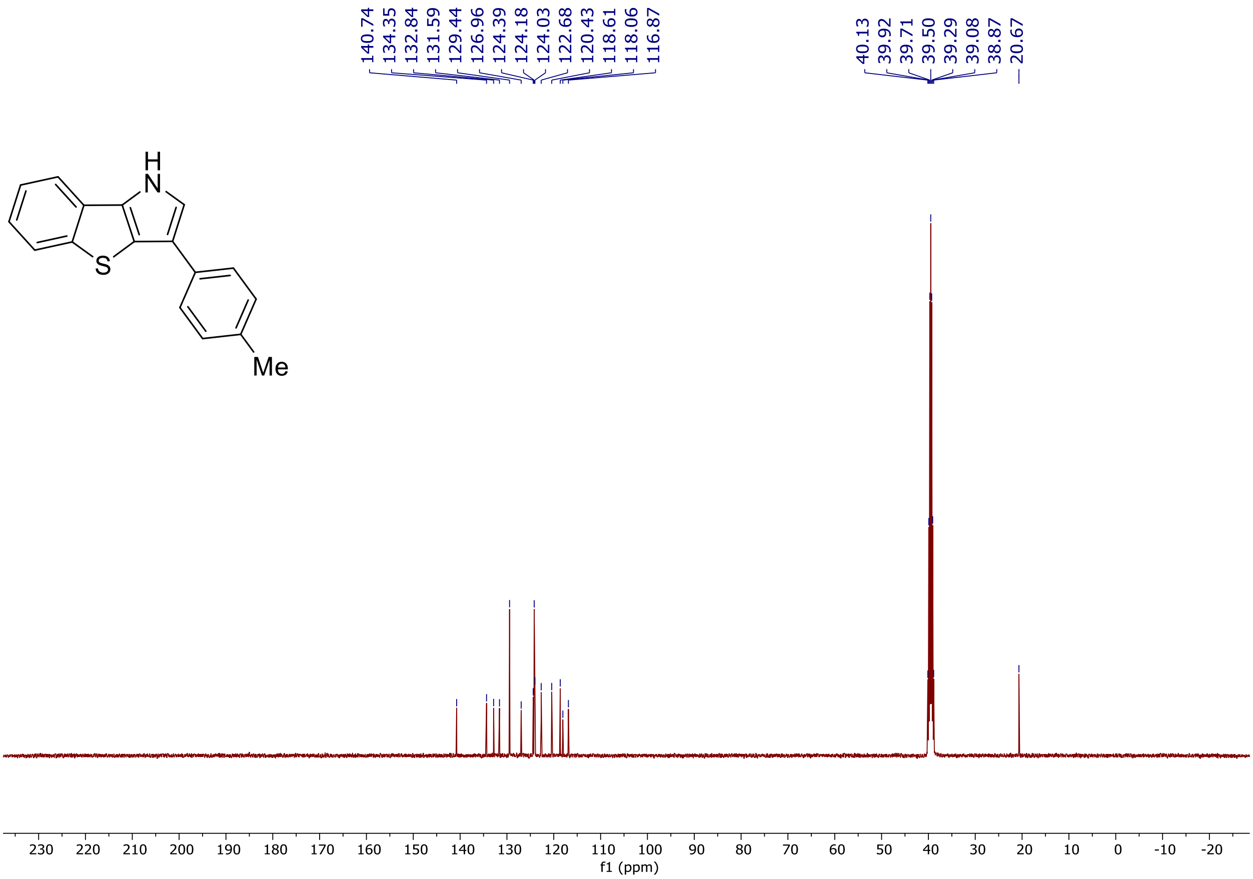

^{1}H NMR spectrum of **3b** (400 MHz, DMSO-d_6)

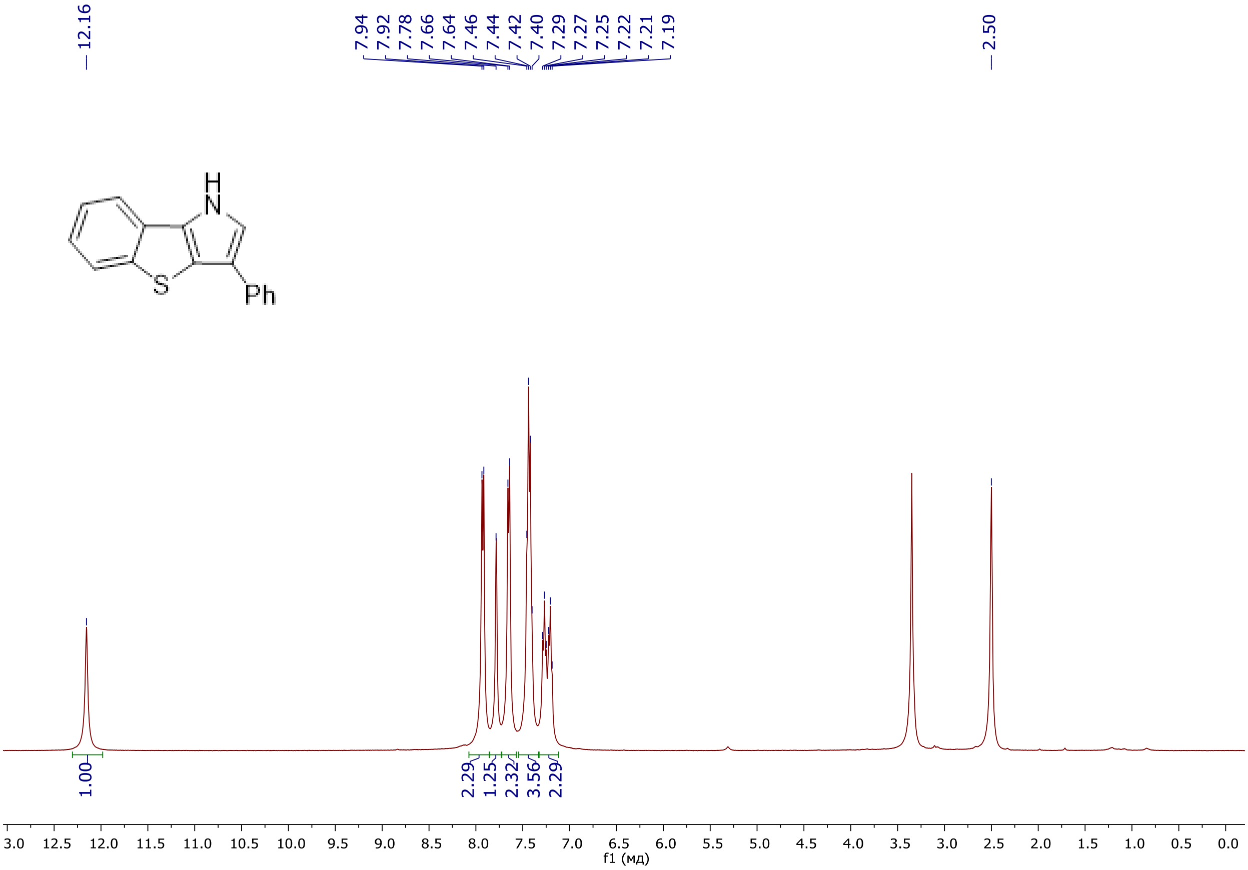

^{13}C{^{1}H} NMR spectrum of **3b** (101 MHz, DMSO-d_6)

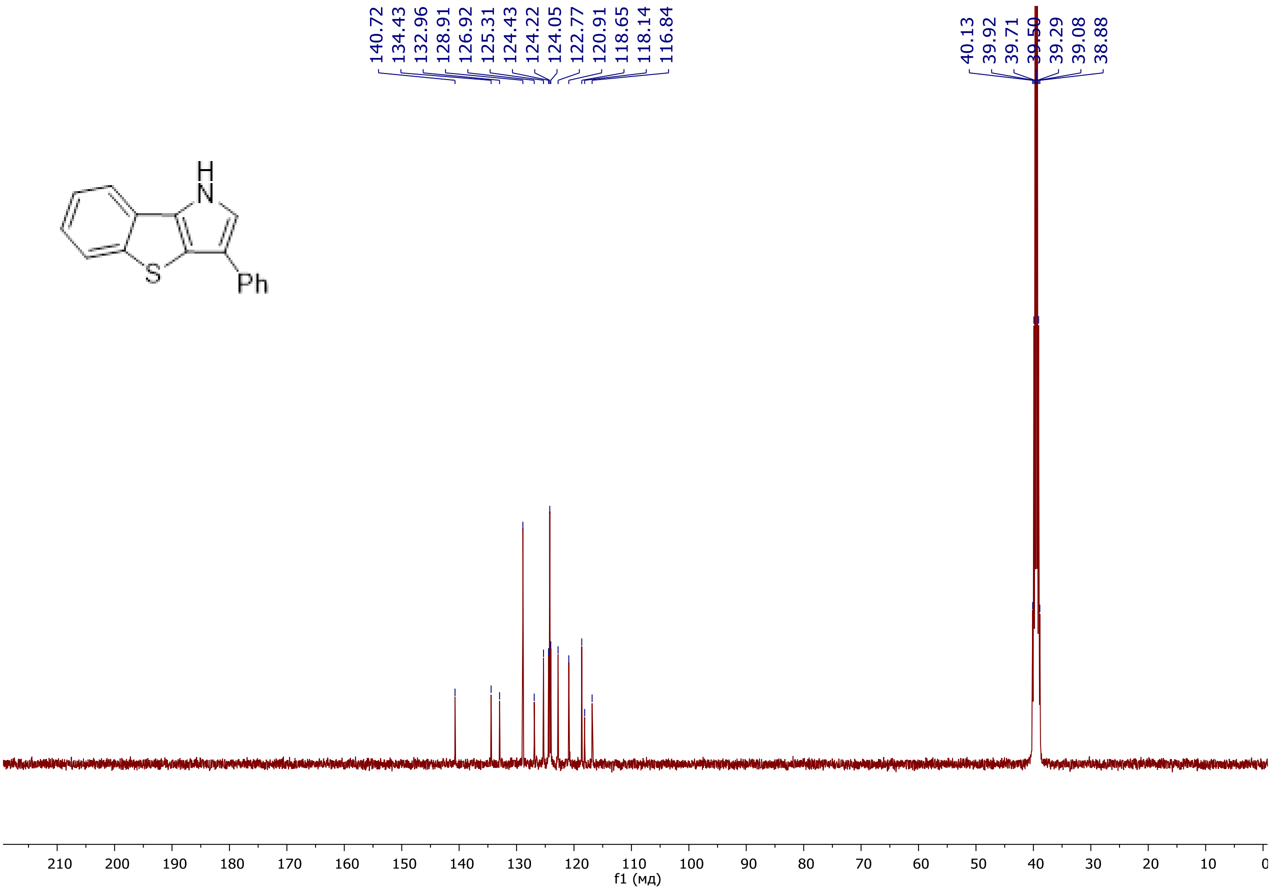

^{1}H NMR spectrum of **3c** (400 MHz, DMSO-d_6)

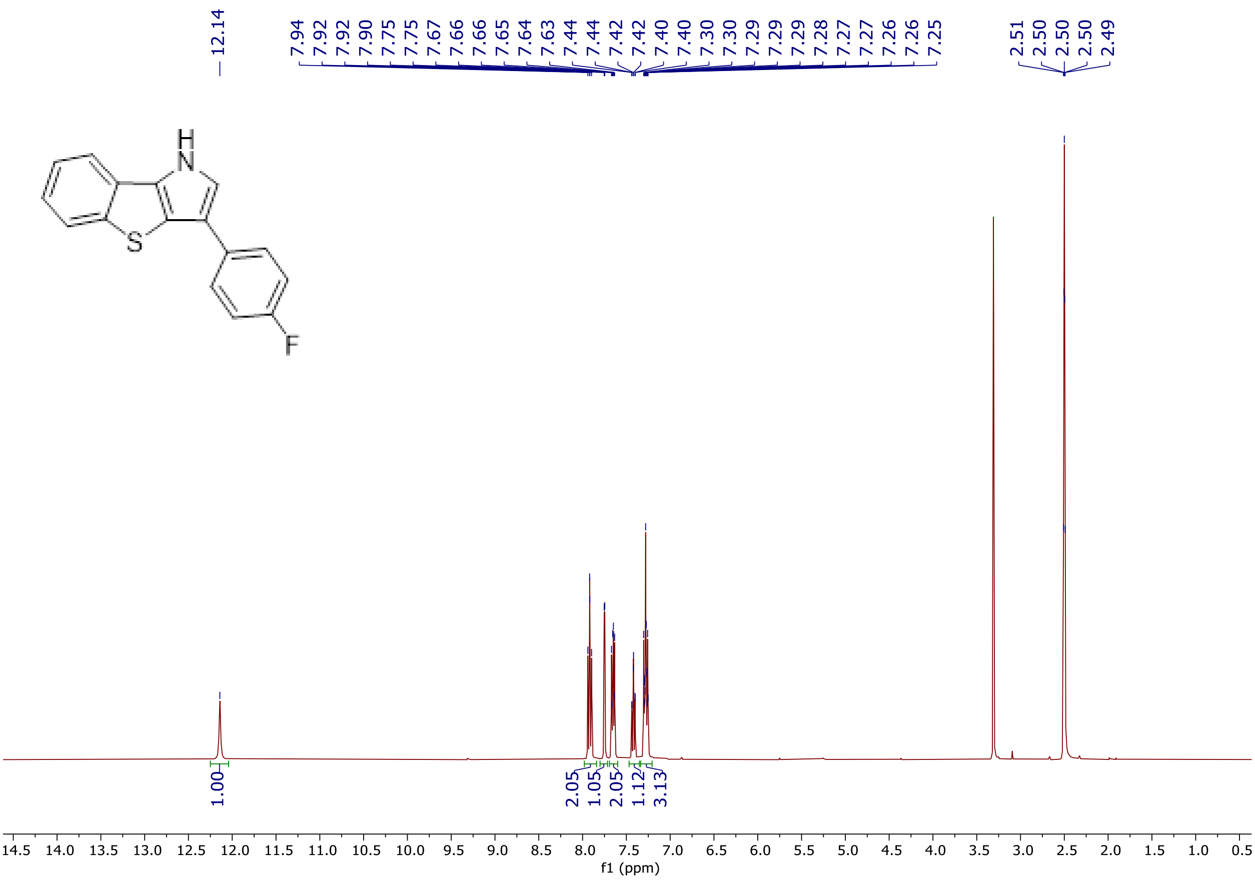

$^{13}C\{^{1}H\}$ NMR spectrum of **3c** (101 MHz, DMSO-d_6)

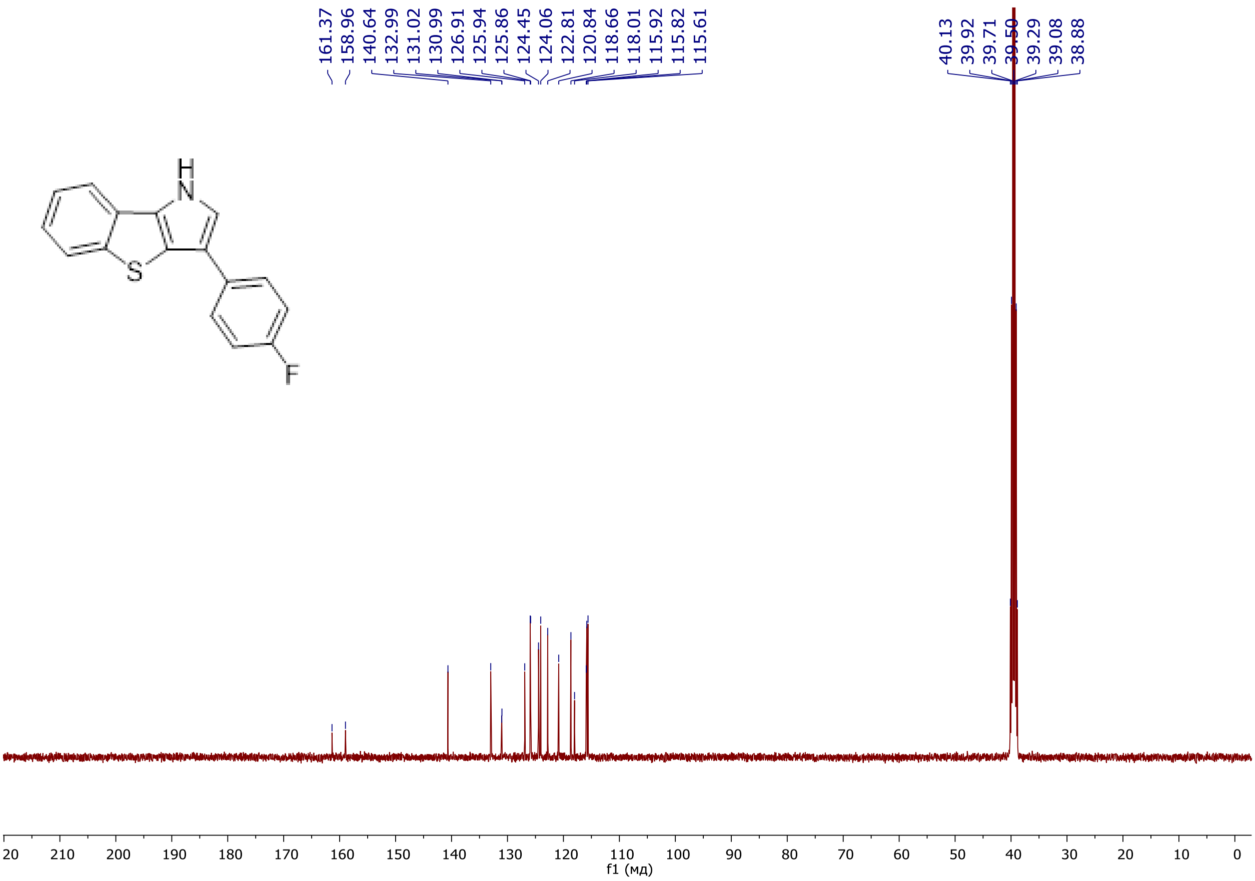

^{1}H NMR spectrum of **3d** (400 MHz, DMSO-d_6)

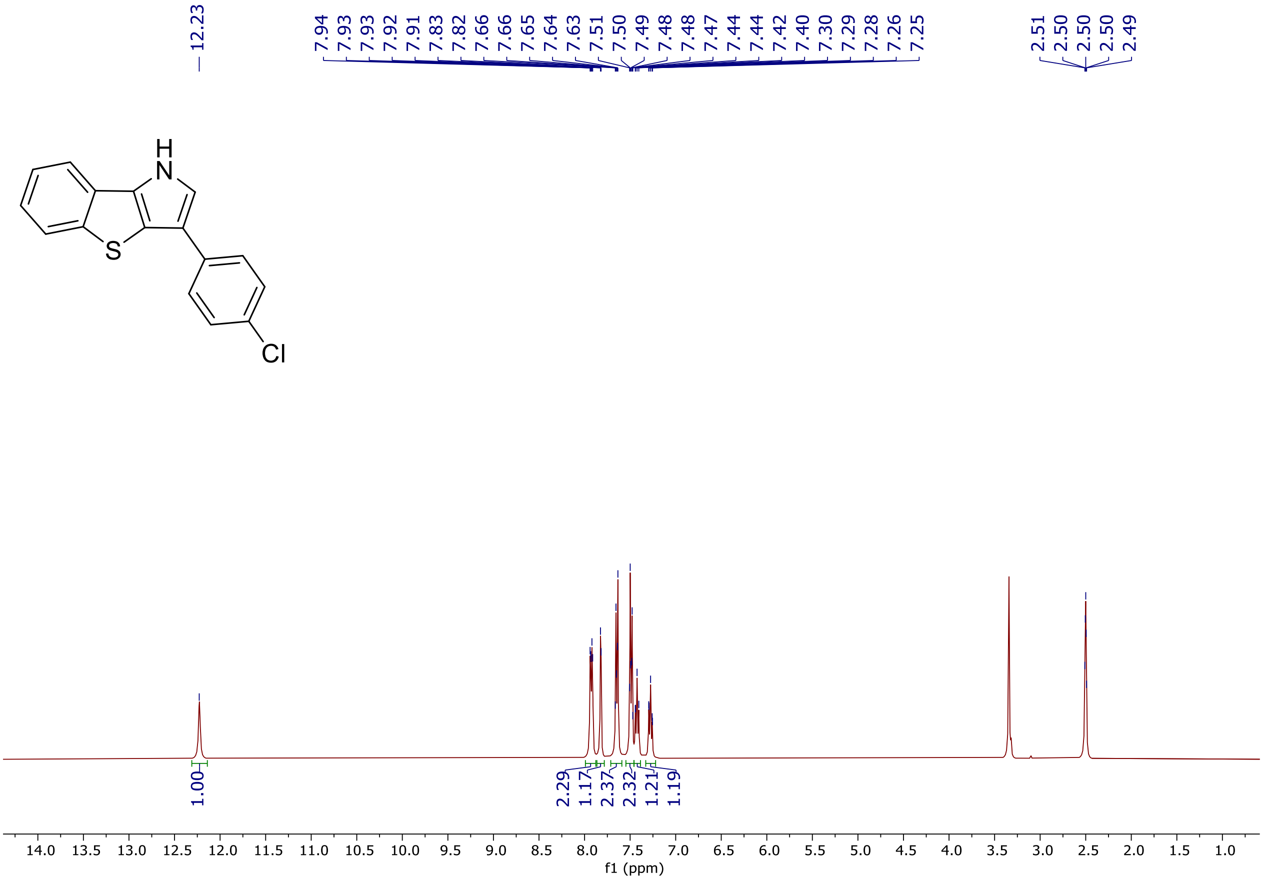

$^{13}C\{^{1}H\}$ NMR spectrum of **3d** (101 MHz, DMSO-d_6)

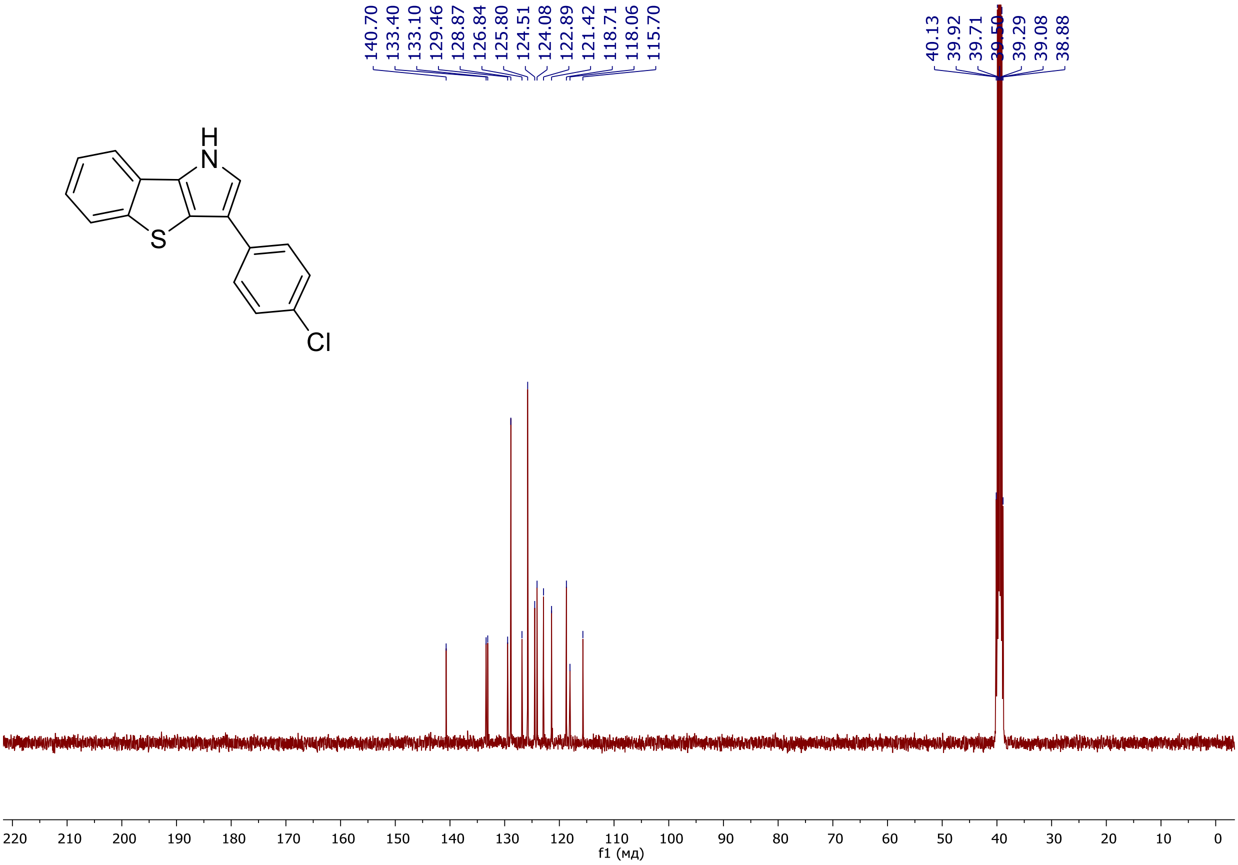

^{1}H NMR spectrum of **3e** (400 MHz, DMSO-d_6)

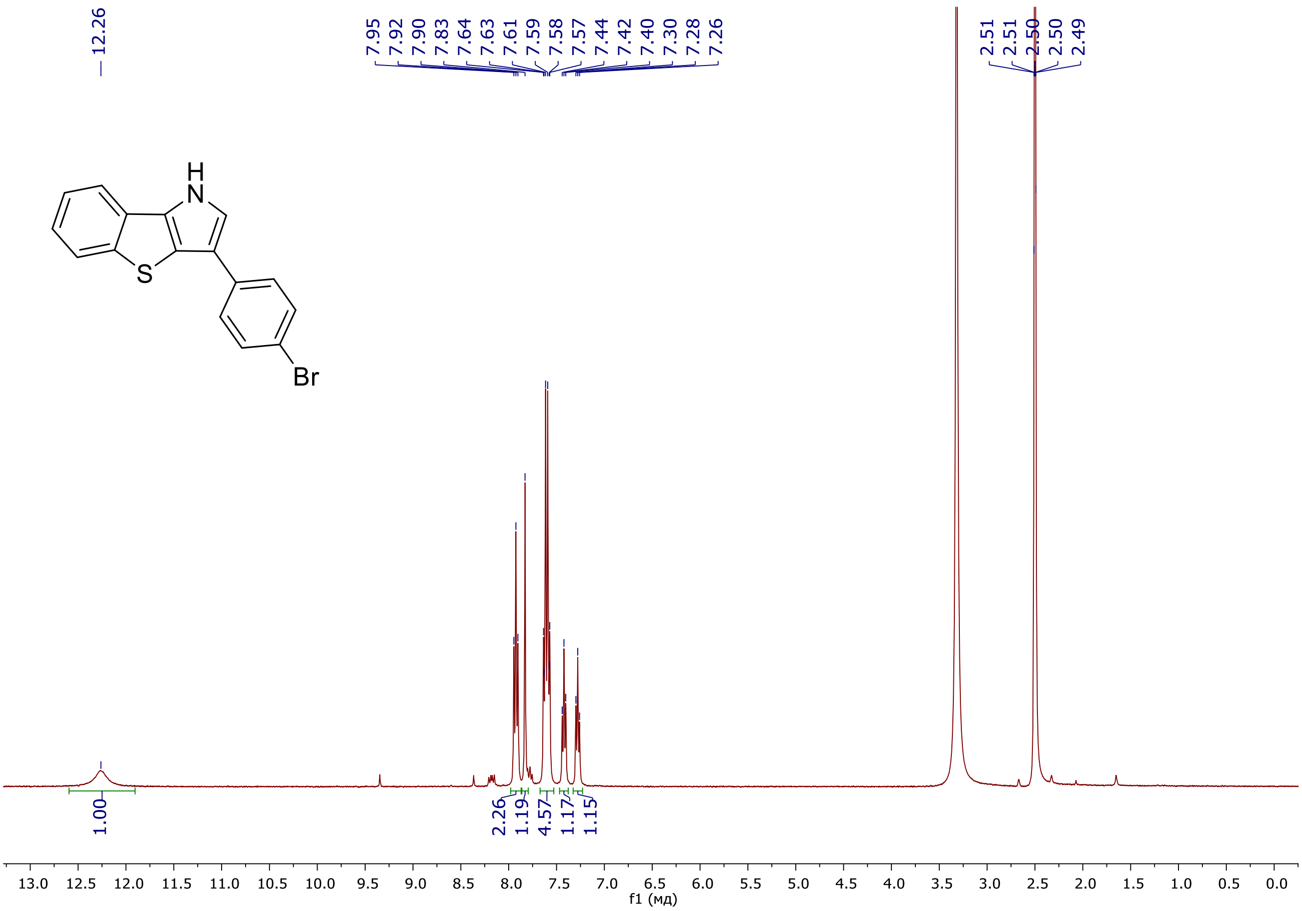

^{13}C{^{1}H} NMR spectrum of **3e** (101 MHz, DMSO-d_6)

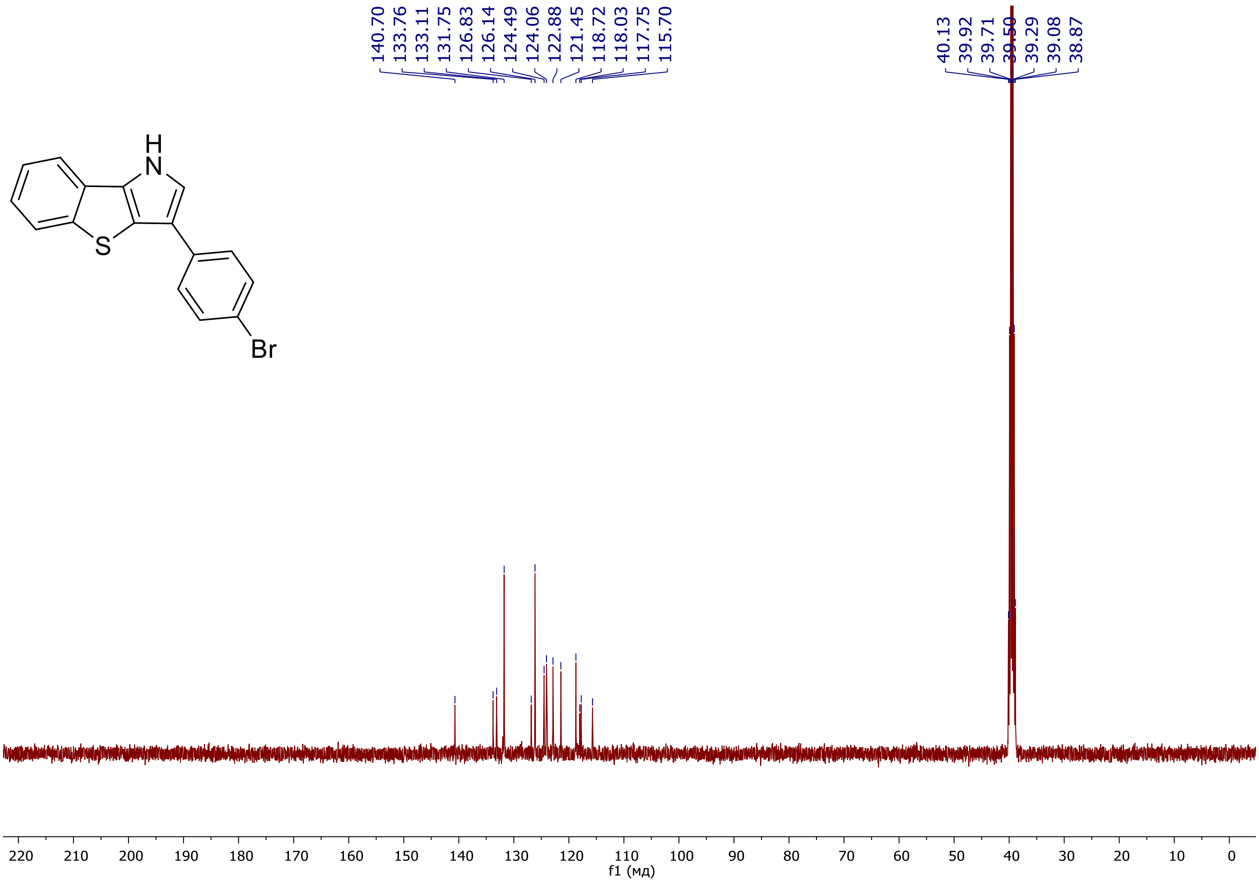

^{1}H NMR spectrum of **3f** (400 MHz, DMSO-d_6)

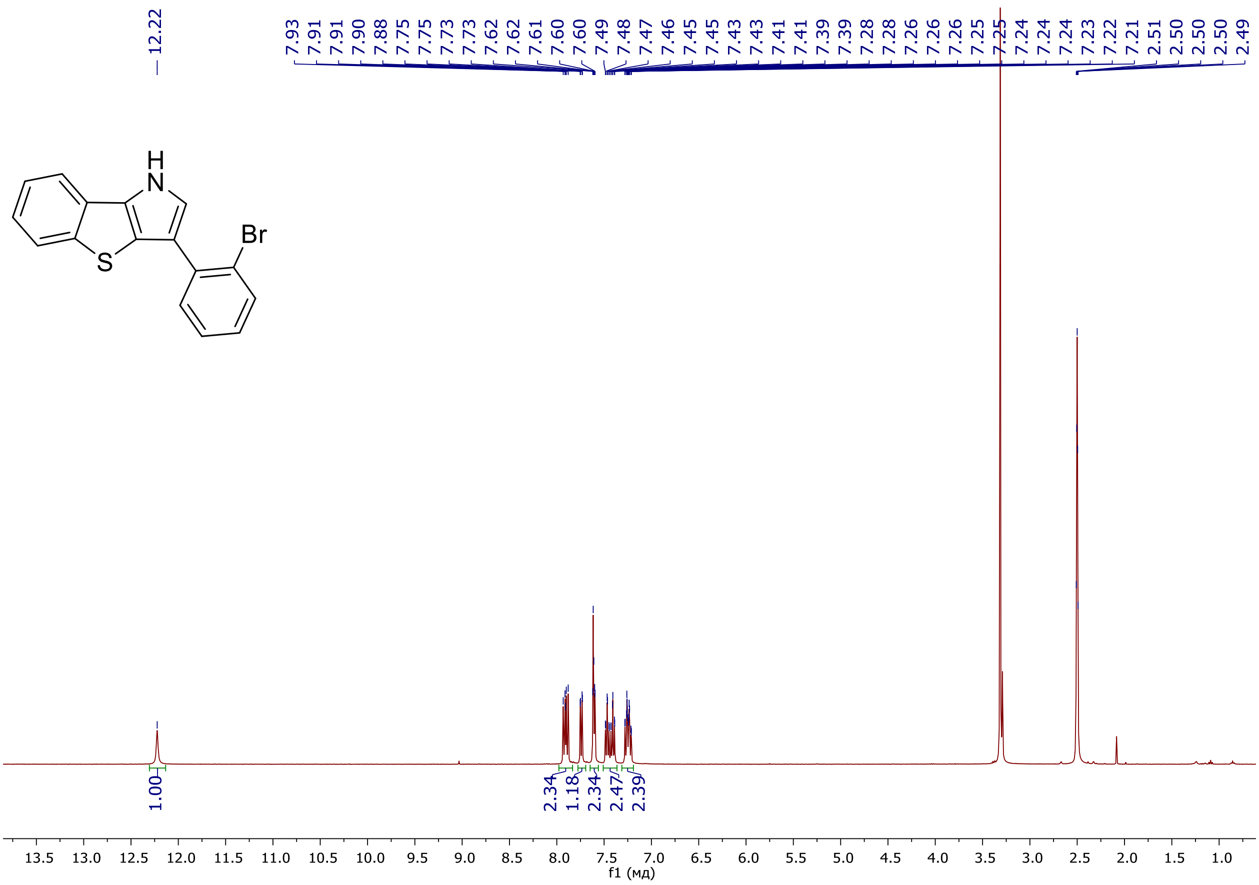

$^{13}C\{^{1}H\}$ NMR spectrum of **3f** (101 MHz, DMSO-d_6)

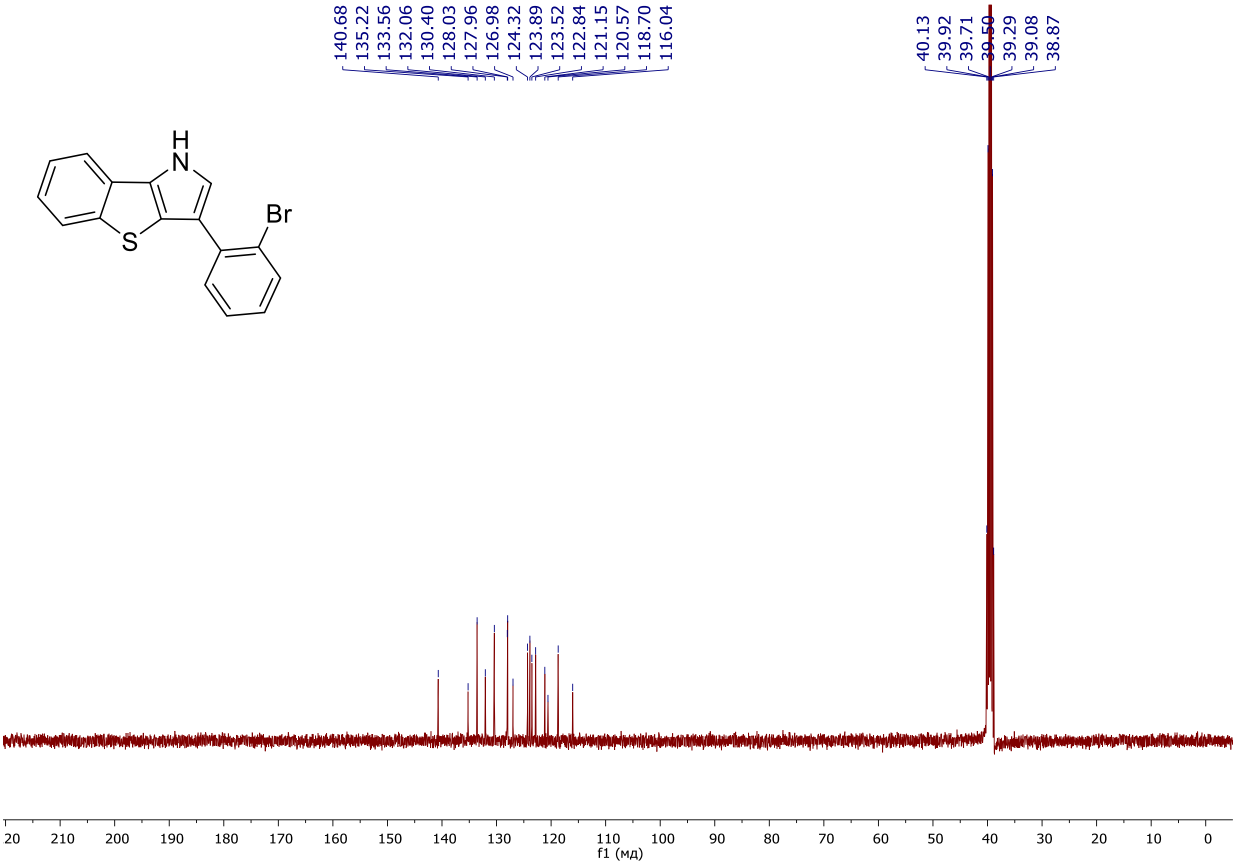

^{1}H NMR spectrum of **3g** (400 MHz, DMSO-d_6)

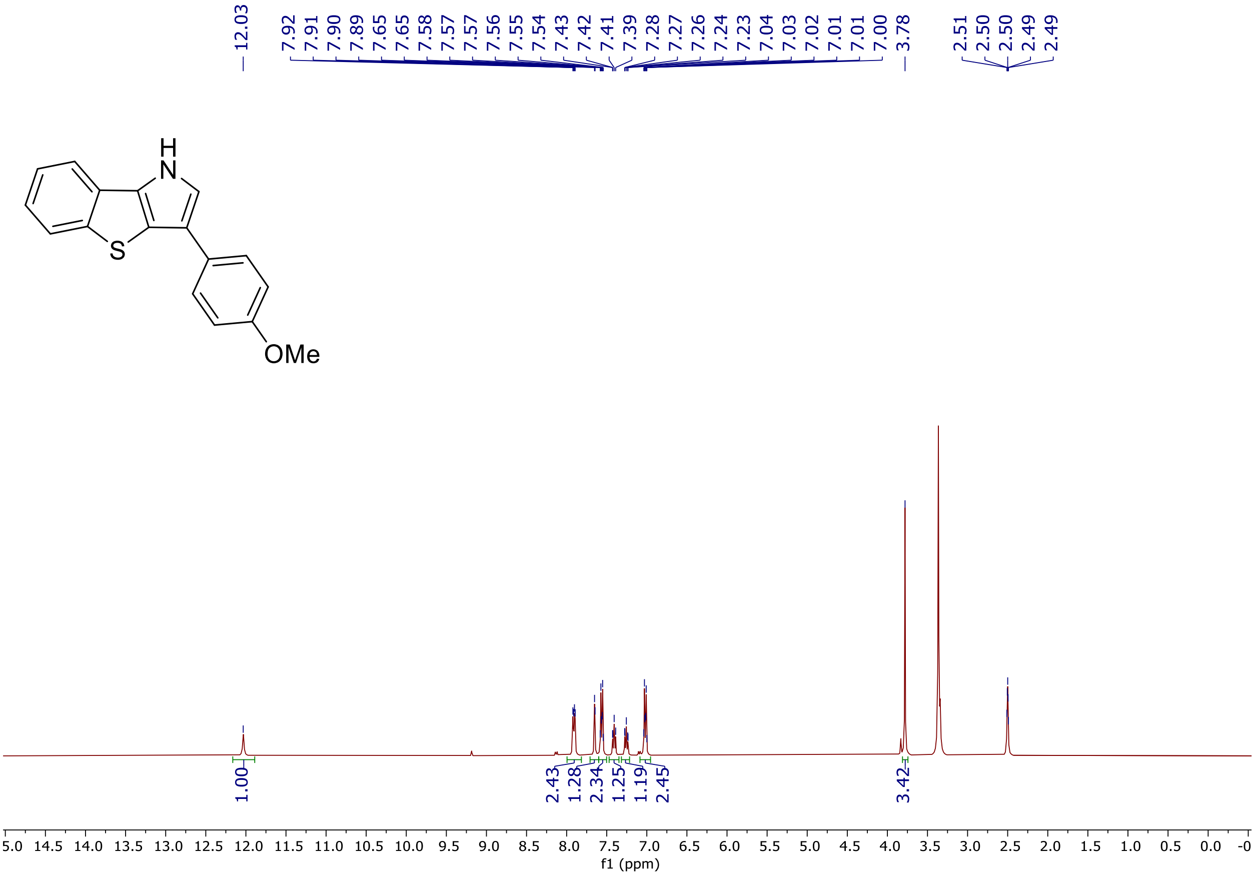

^{13}C{^{1}H} NMR spectrum of **3g** (101 MHz, DMSO-d_6)

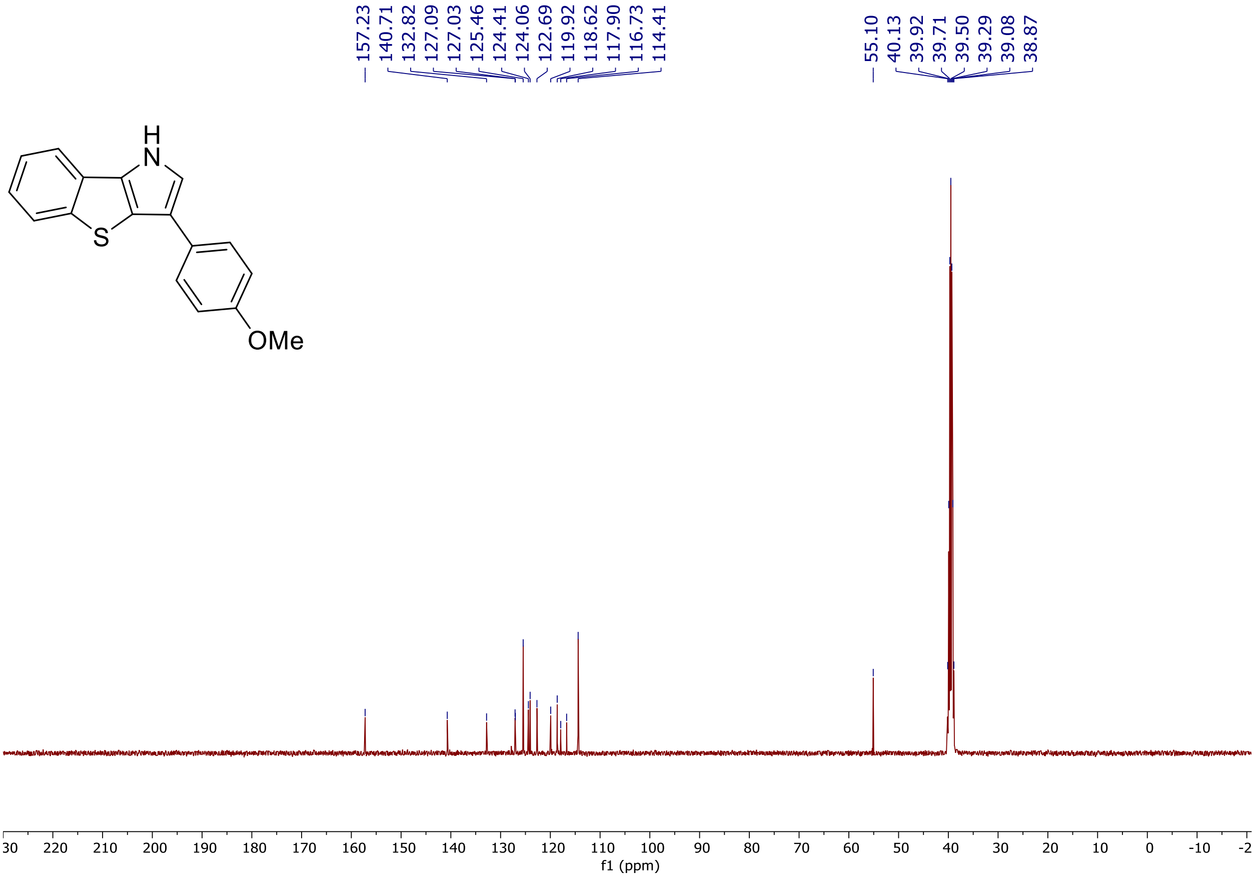

^{1}H NMR spectrum of **3h** (400 MHz, DMSO-d_6)

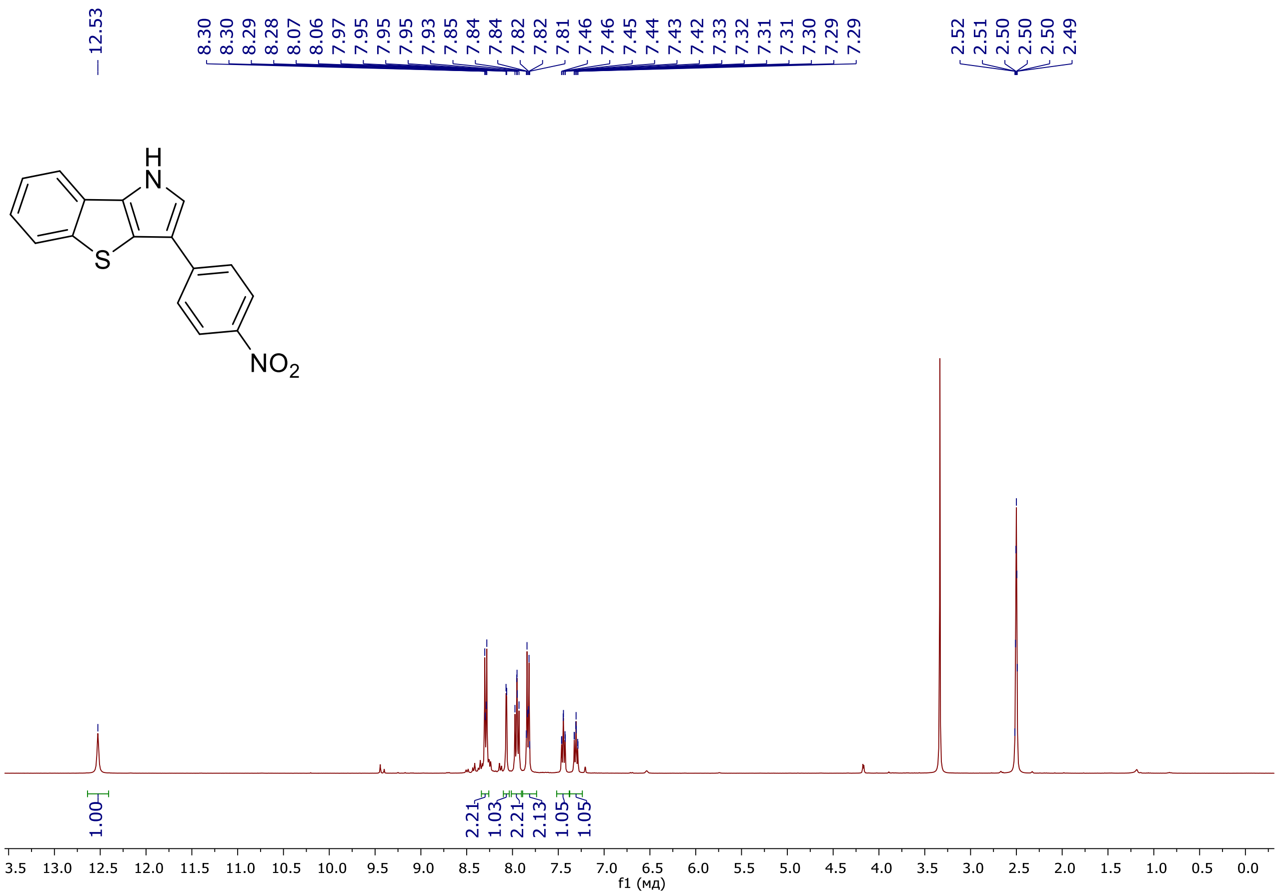

^{13}C{^{1}H} NMR spectrum of **3h** (101 MHz, DMSO-d_6)

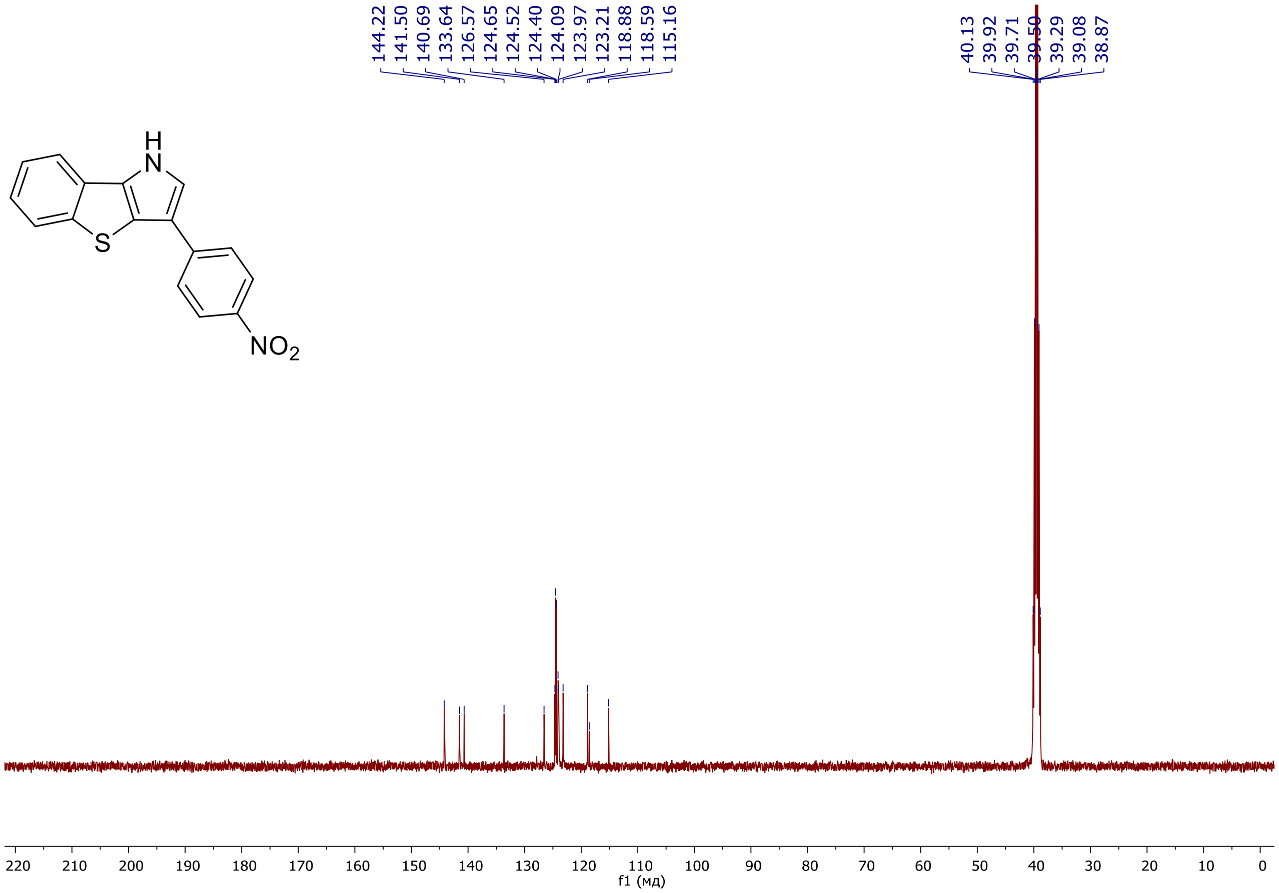

^{1}H NMR spectrum of **3i** (400 MHz, DMSO-d_6)

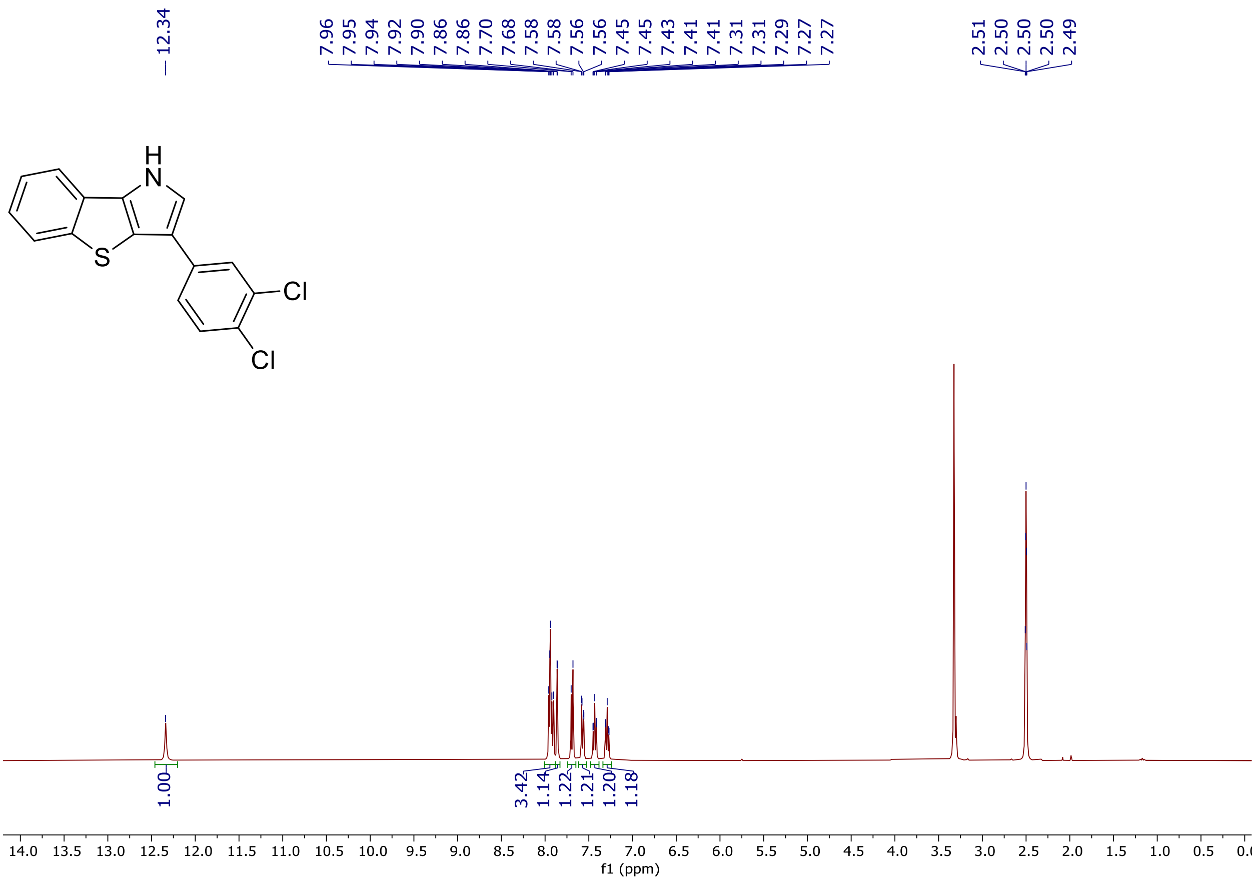

^{13}C{^{1}H} NMR spectrum of **3i** (101 MHz, DMSO-d_6)

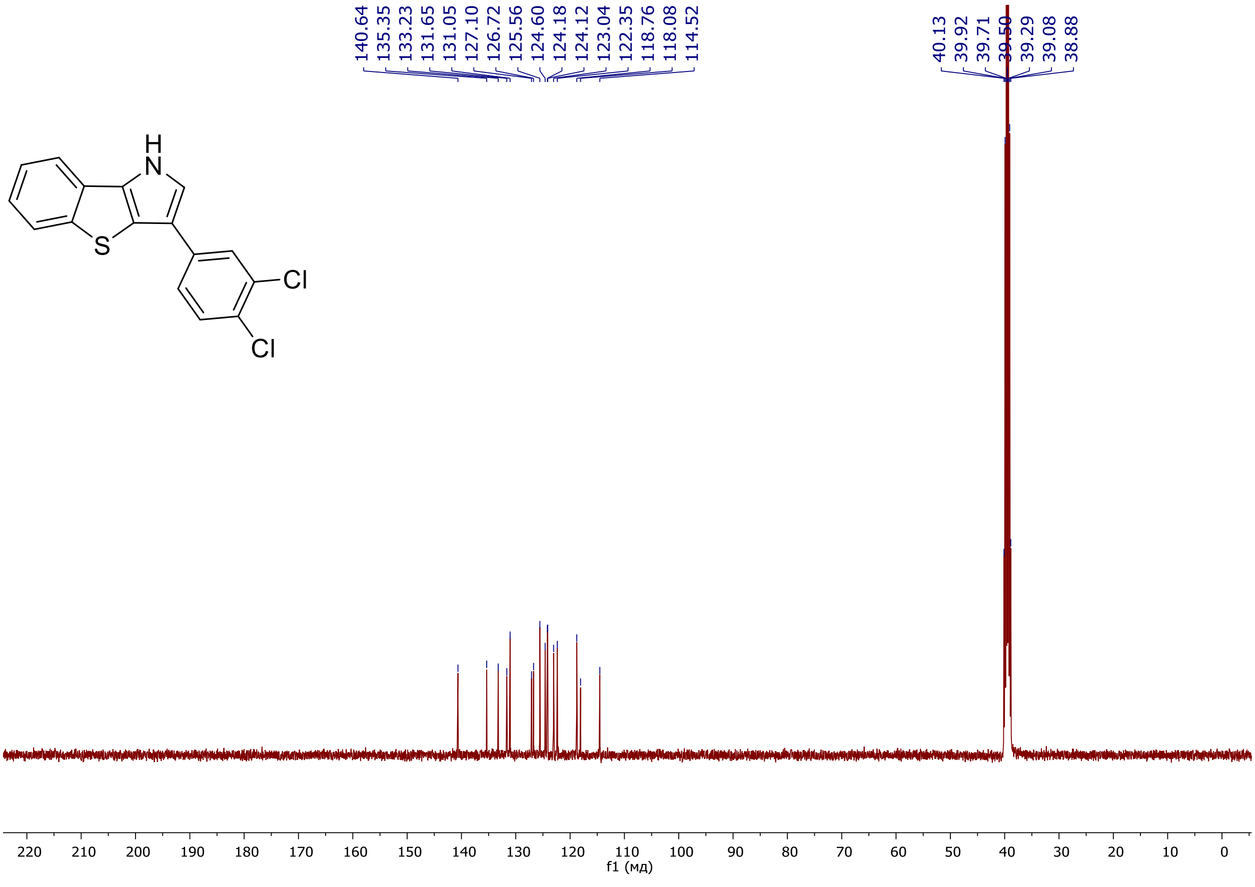

^{1}H NMR spectrum of **3j** (400 MHz, DMSO-d_6)

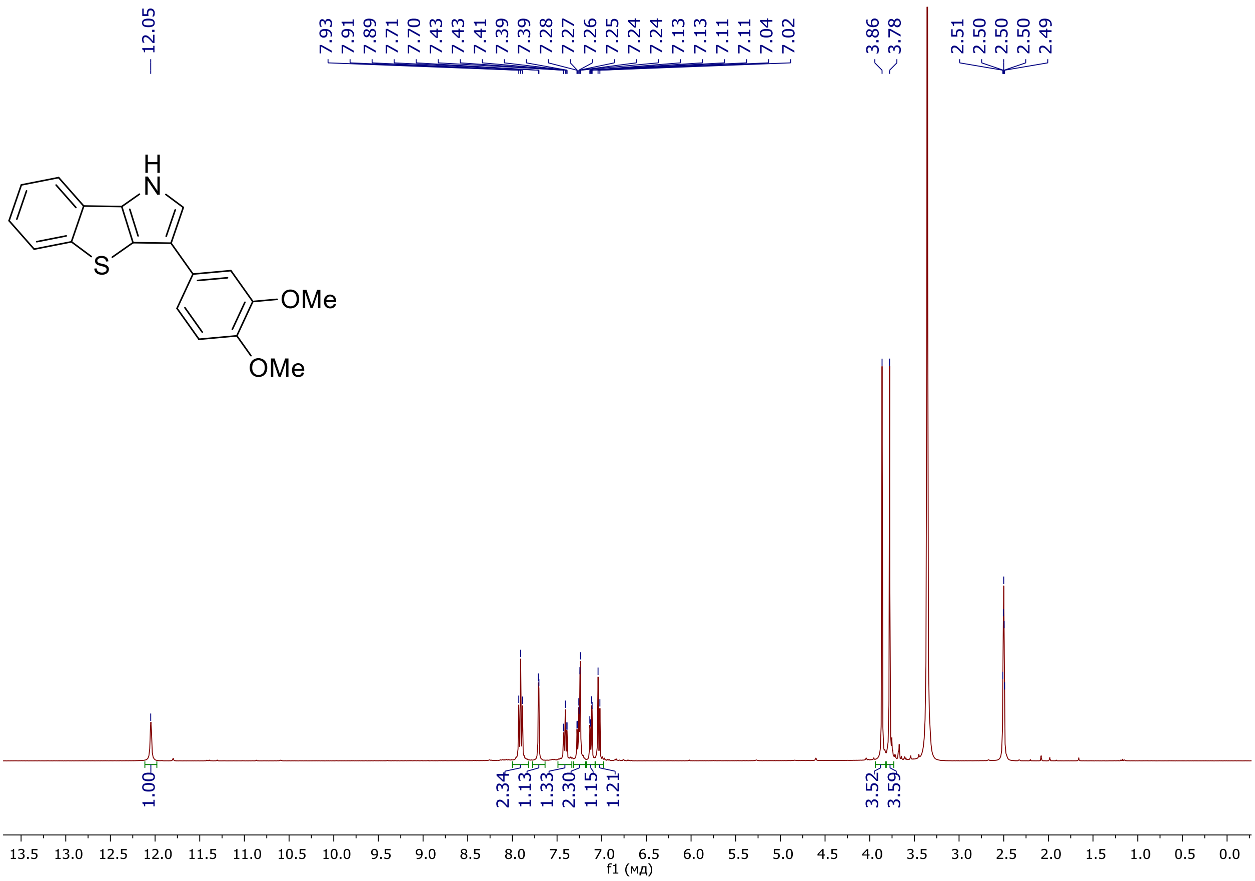

^{13}C{^{1}H} NMR spectrum of **3j** (101 MHz, DMSO-d_6)

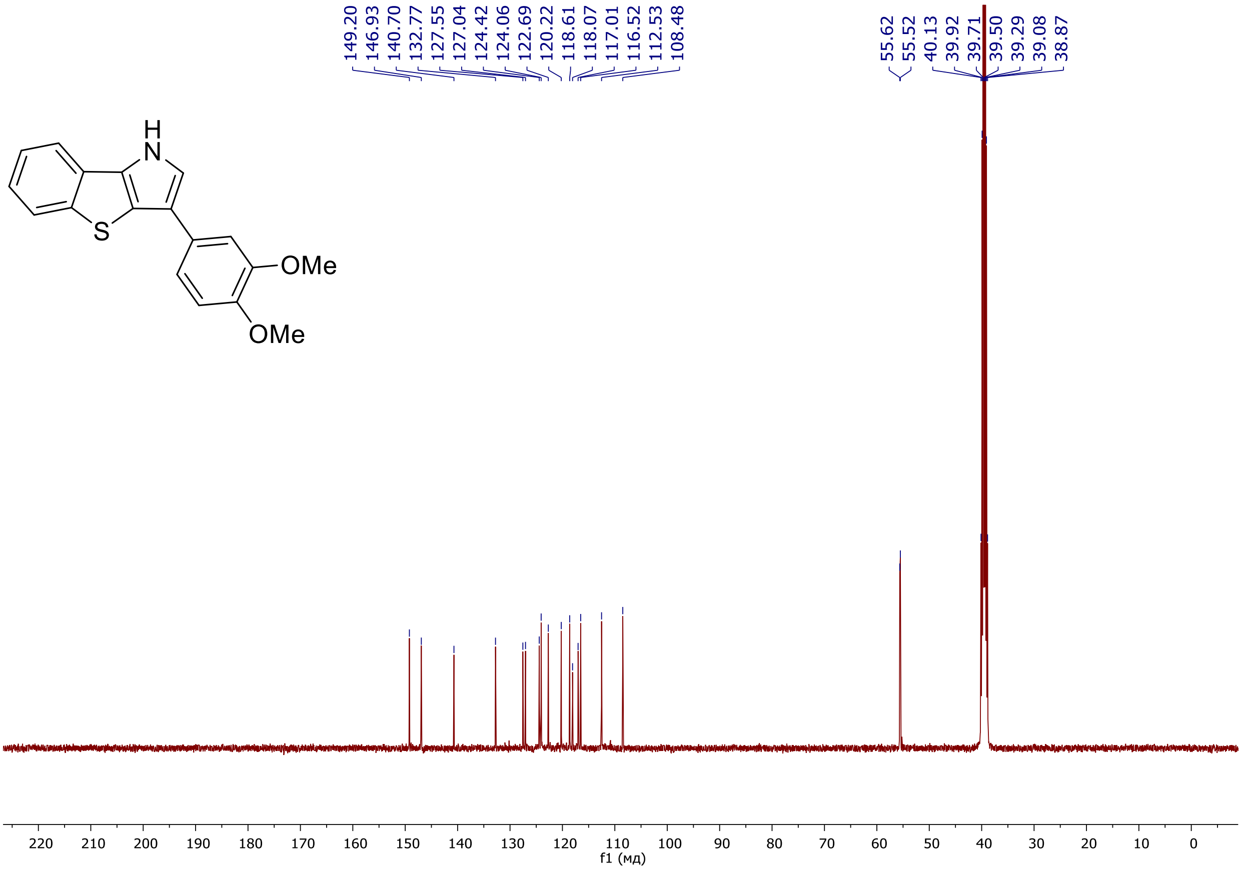

^{1}H NMR spectrum of **3k** (400 MHz, DMSO-d_6)

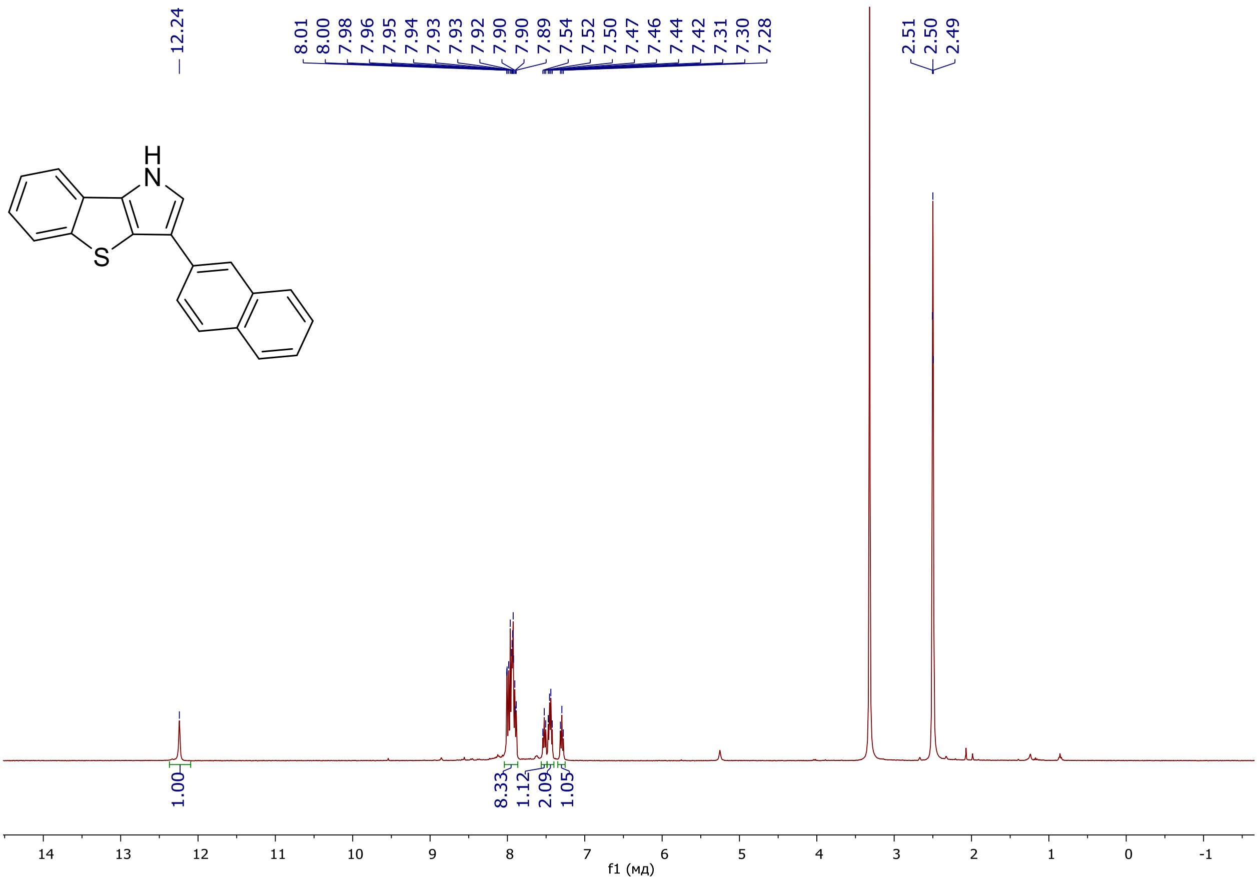

^{13}C{^{1}H} NMR spectrum of **3k** (101 MHz, DMSO-d_6)

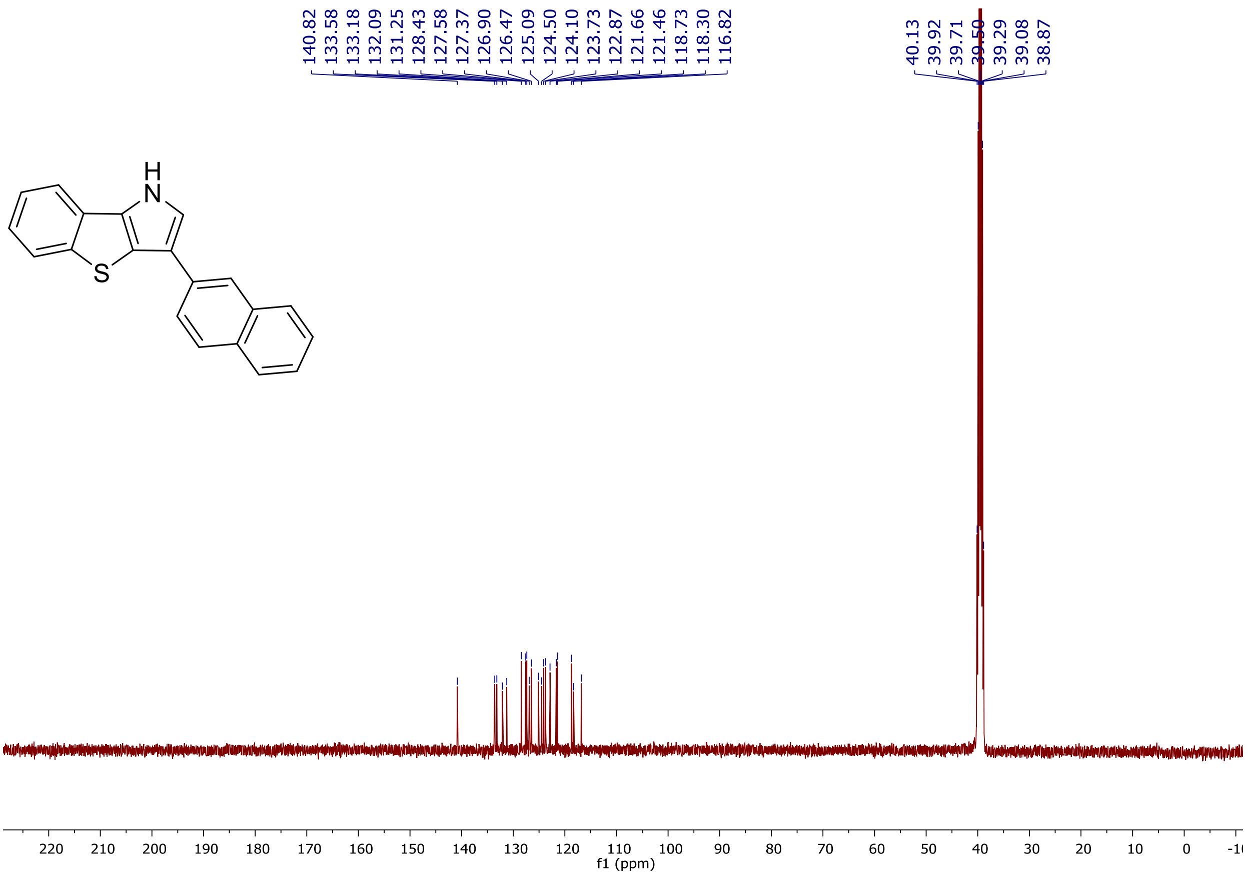

^{1}H NMR spectrum of **6** (400 MHz, DMSO-d_6)

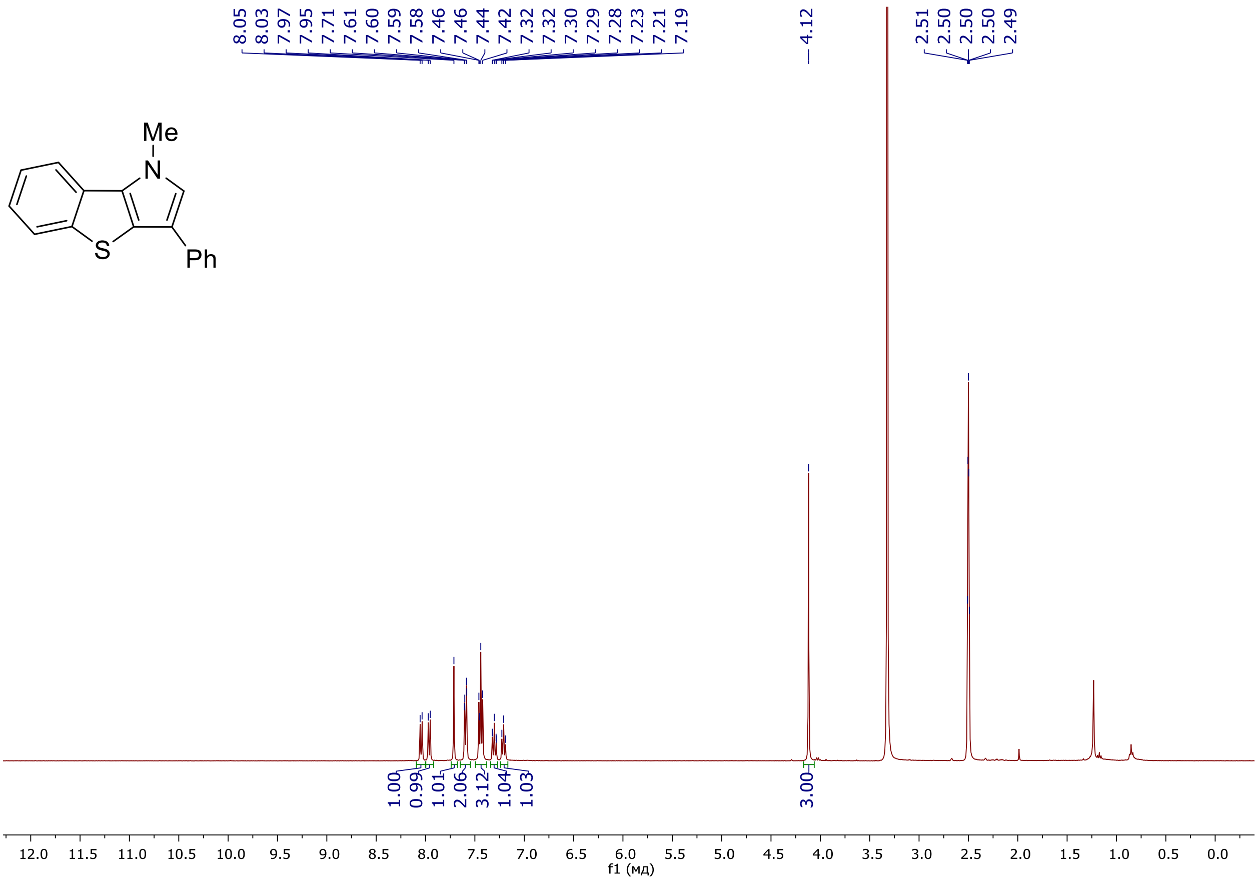

$^{13}C\{^{1}H\}$ NMR spectrum of **6** (101 MHz, DMSO-d_6)

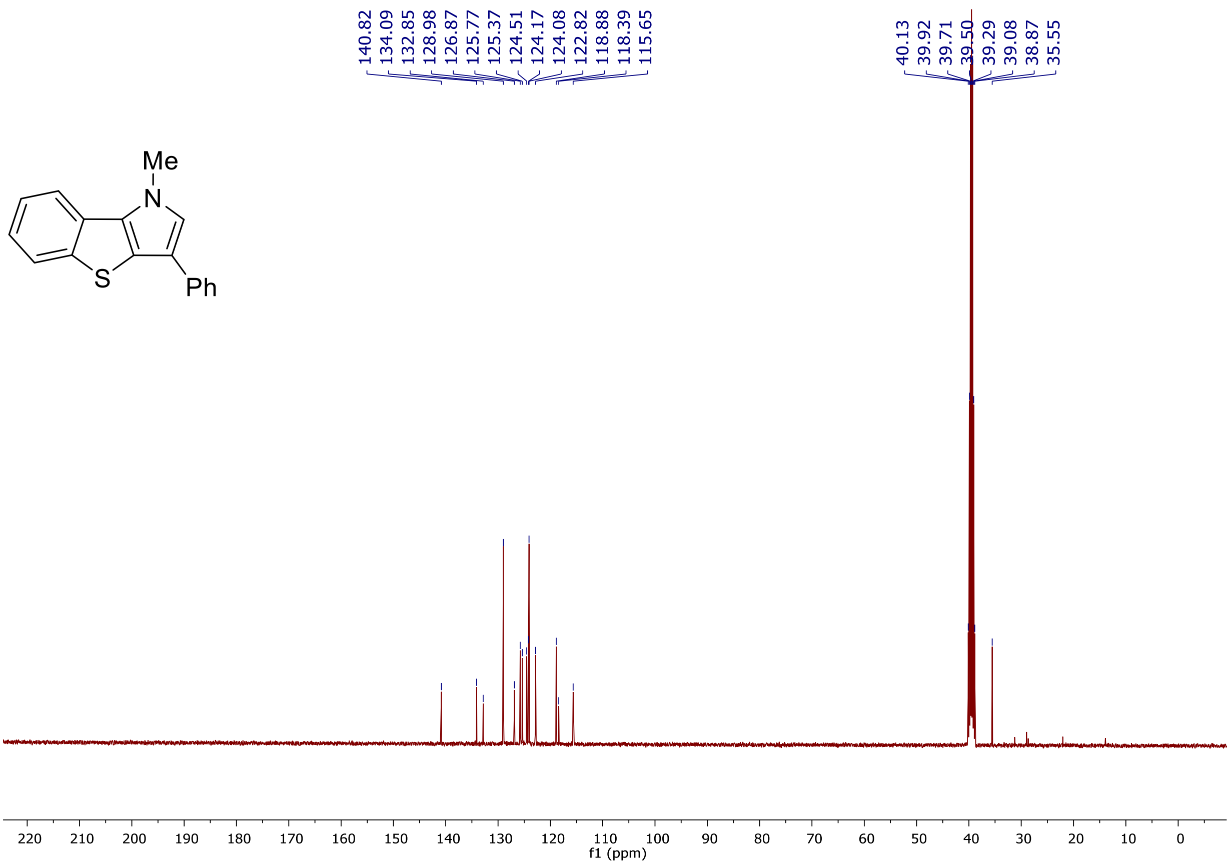

^{1}H NMR spectrum of **7** (400 MHz, DMSO-d_6)

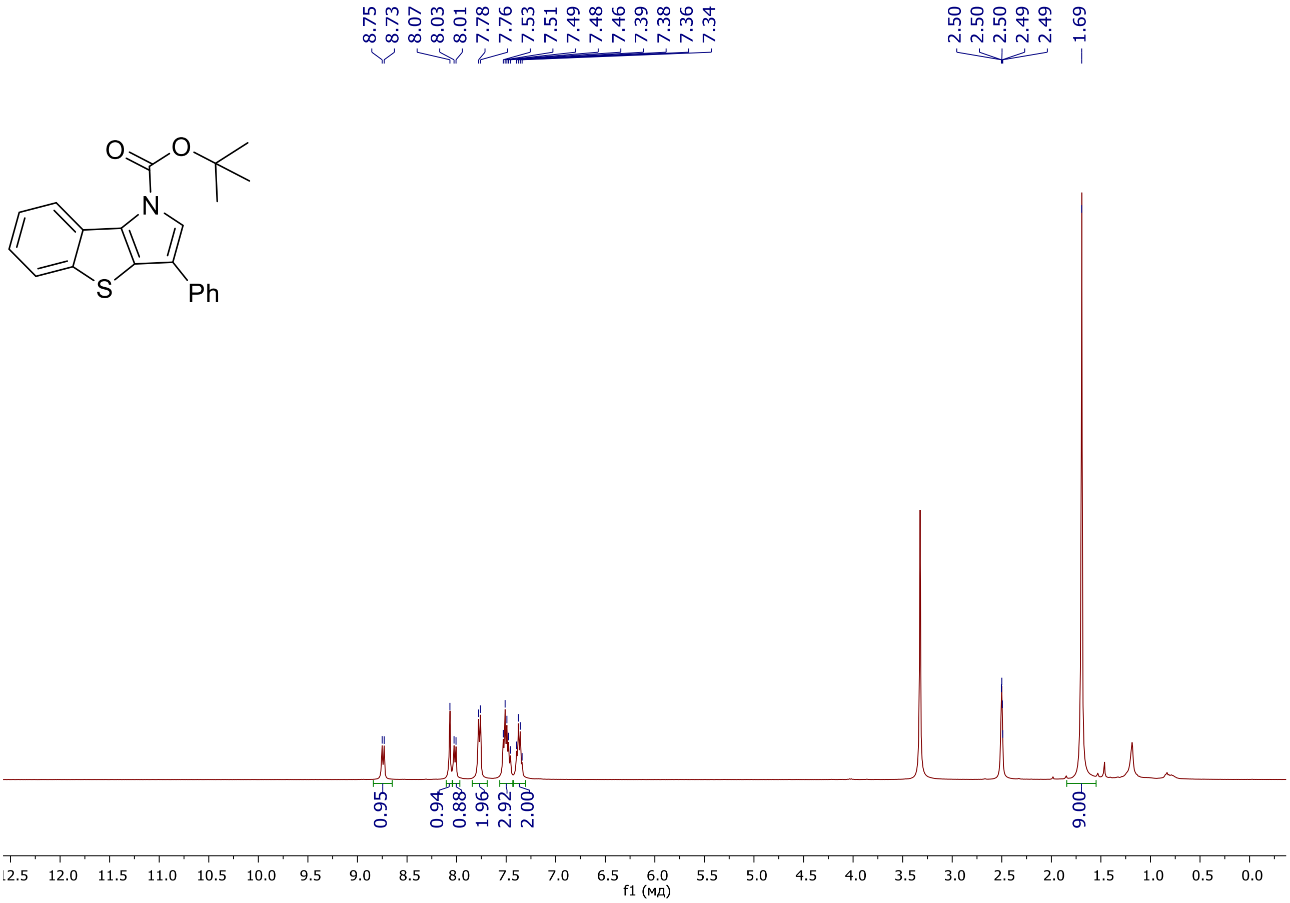

^{13}C{^{1}H} NMR spectrum of **7** (101 MHz, DMSO-d_6)

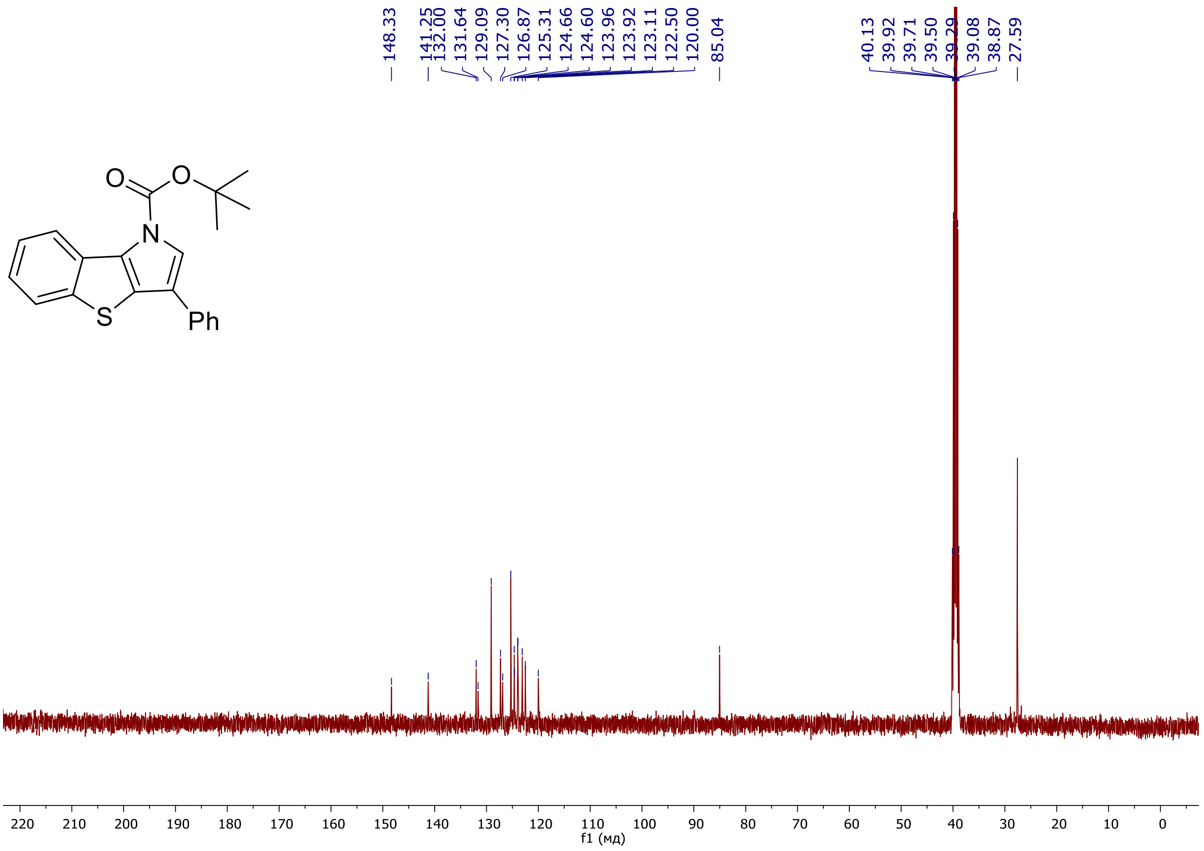

^{1}H NMR spectrum of **8** (400 MHz, DMSO-d_6)

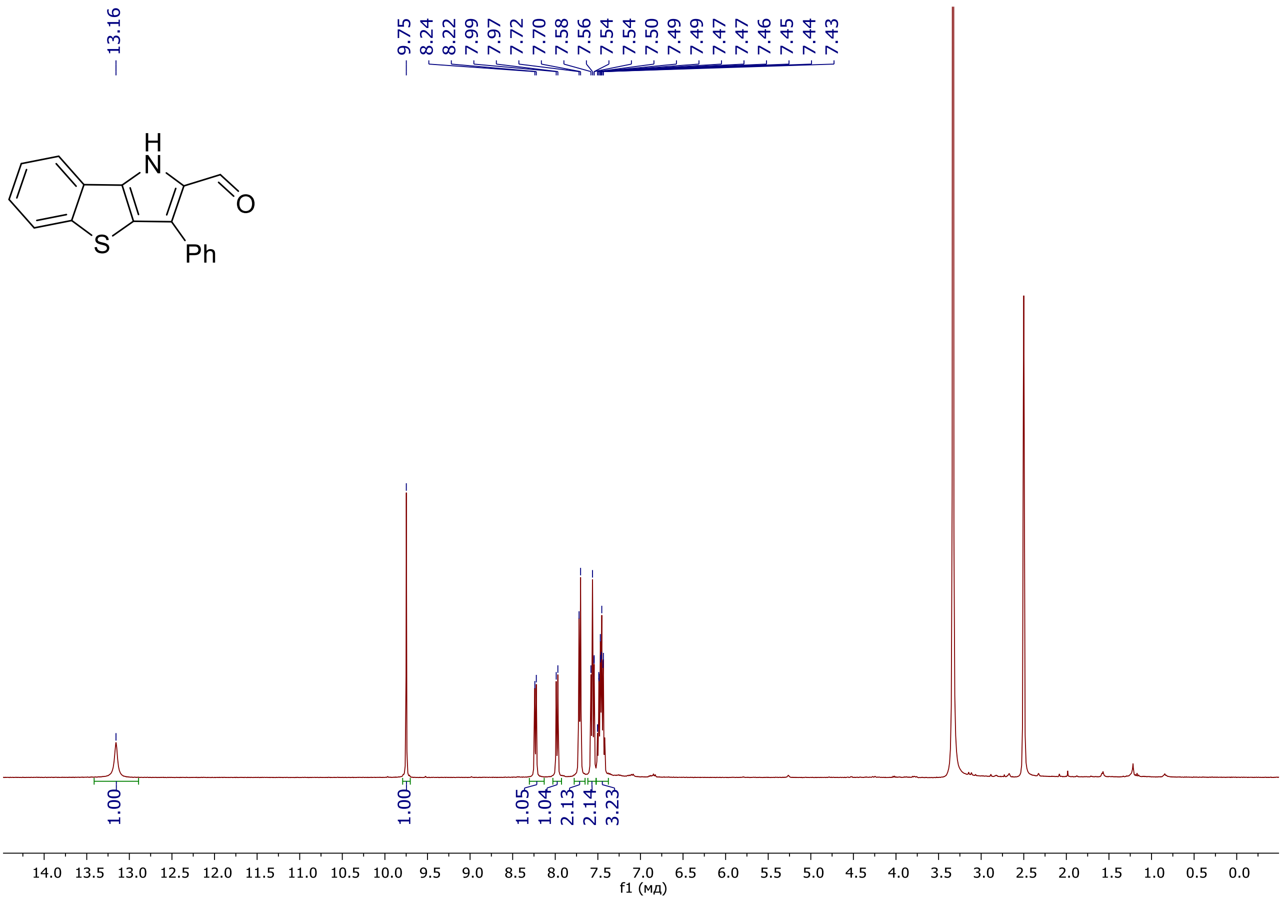

^{13}C{^{1}H} NMR spectrum of **8** (101 MHz, DMSO-d_6)

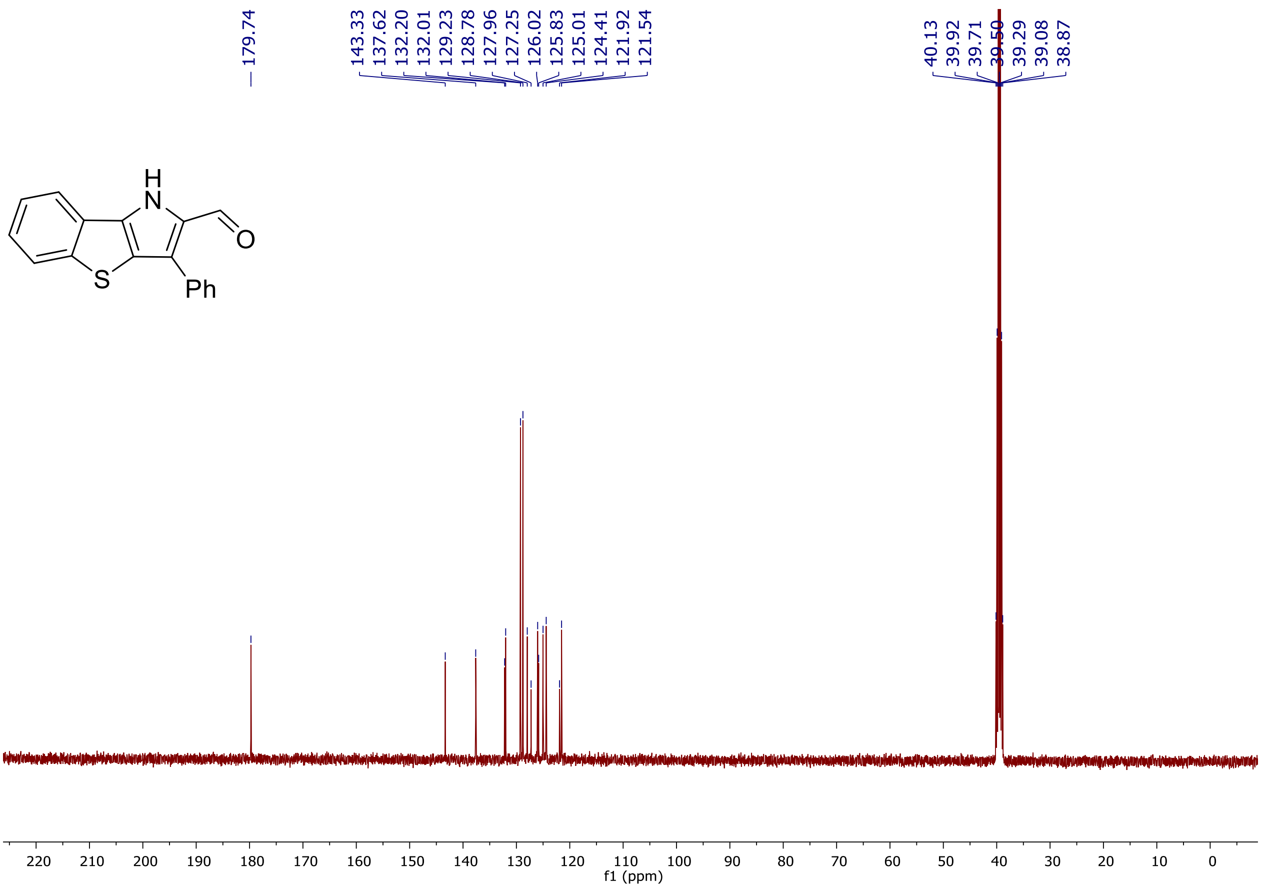

^{1}H NMR spectrum of **10** (400 MHz, $CDCl_3$)

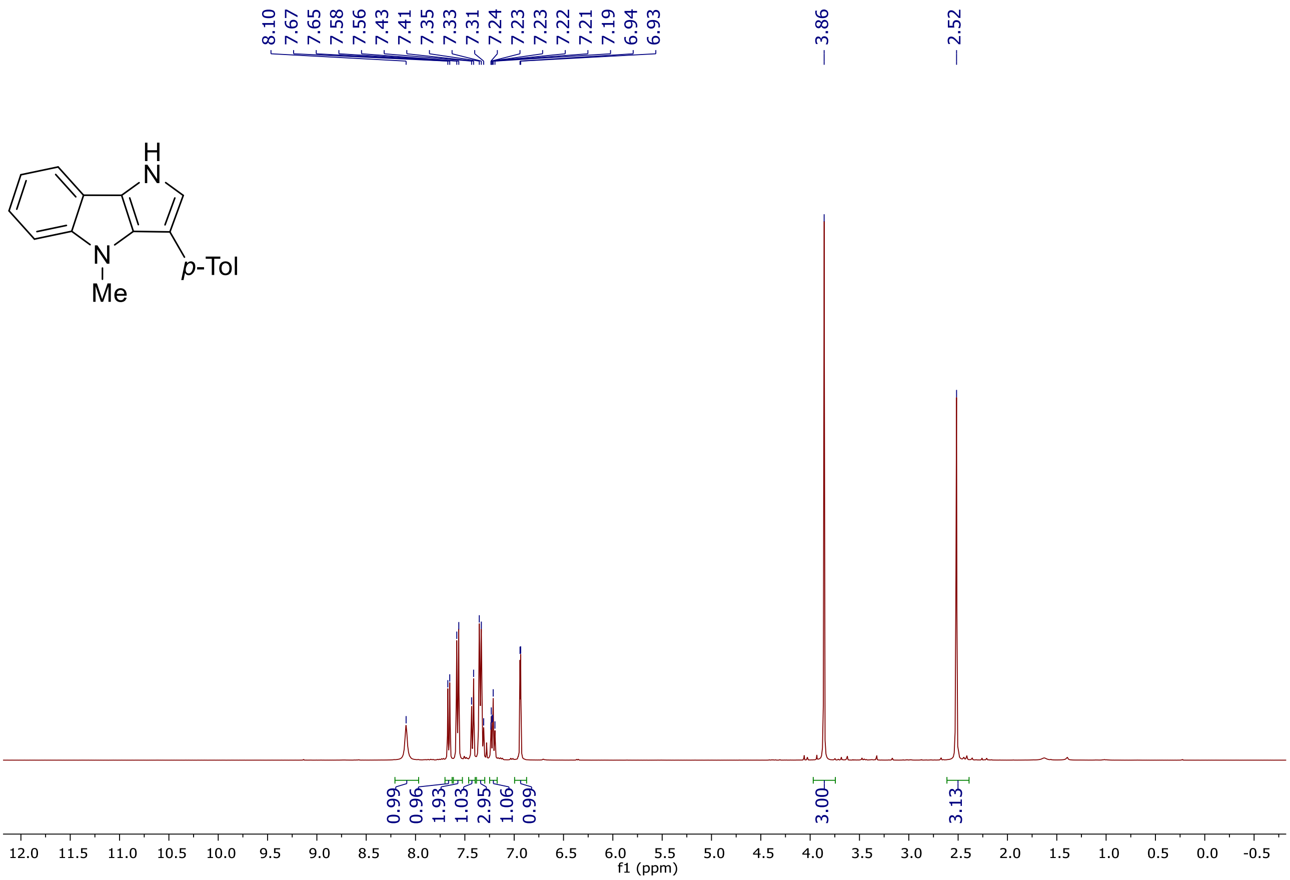

$^{13}C\{^{1}H\}$ NMR spectrum of **10** (101 MHz, $CDCl_3$)

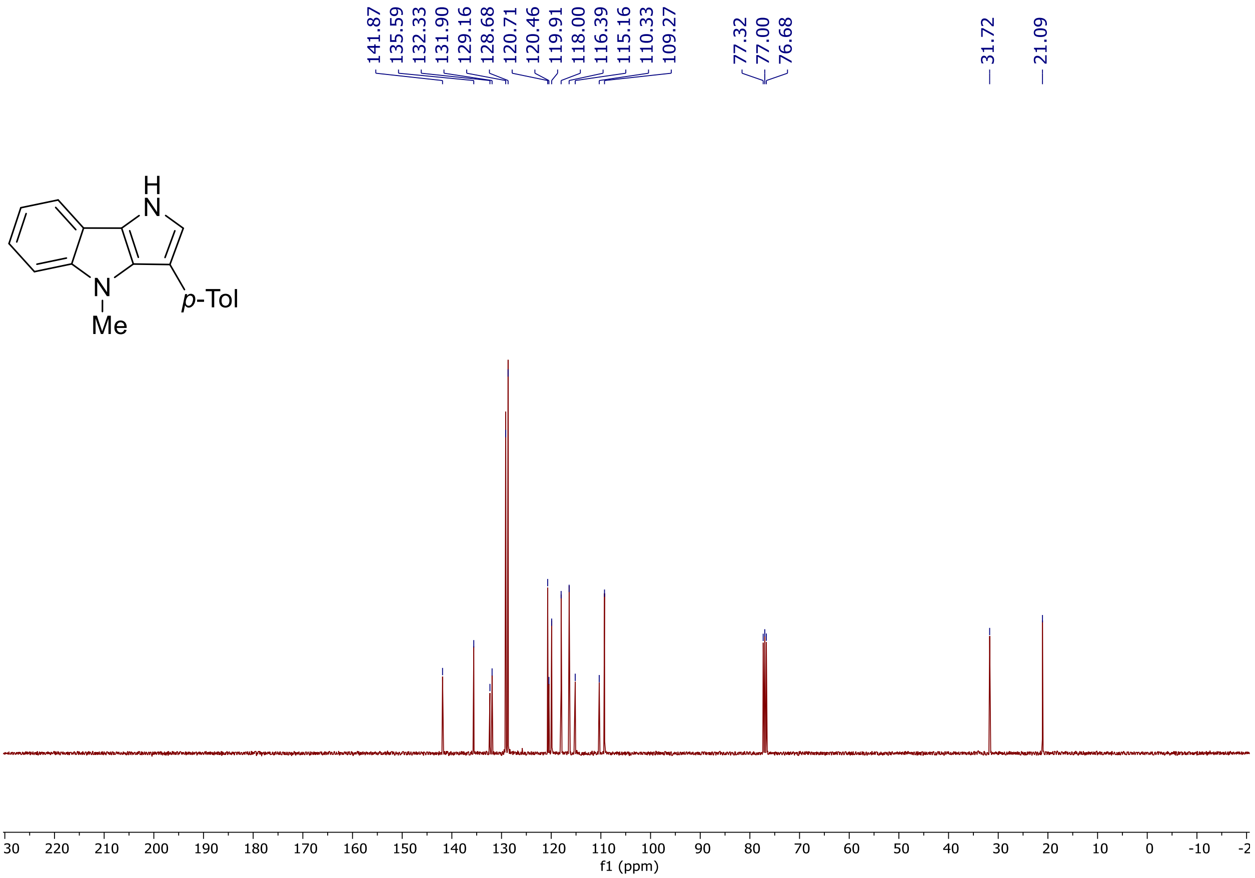

^{1}H NMR spectrum of **11** (400 MHz, $CDCl_3$)

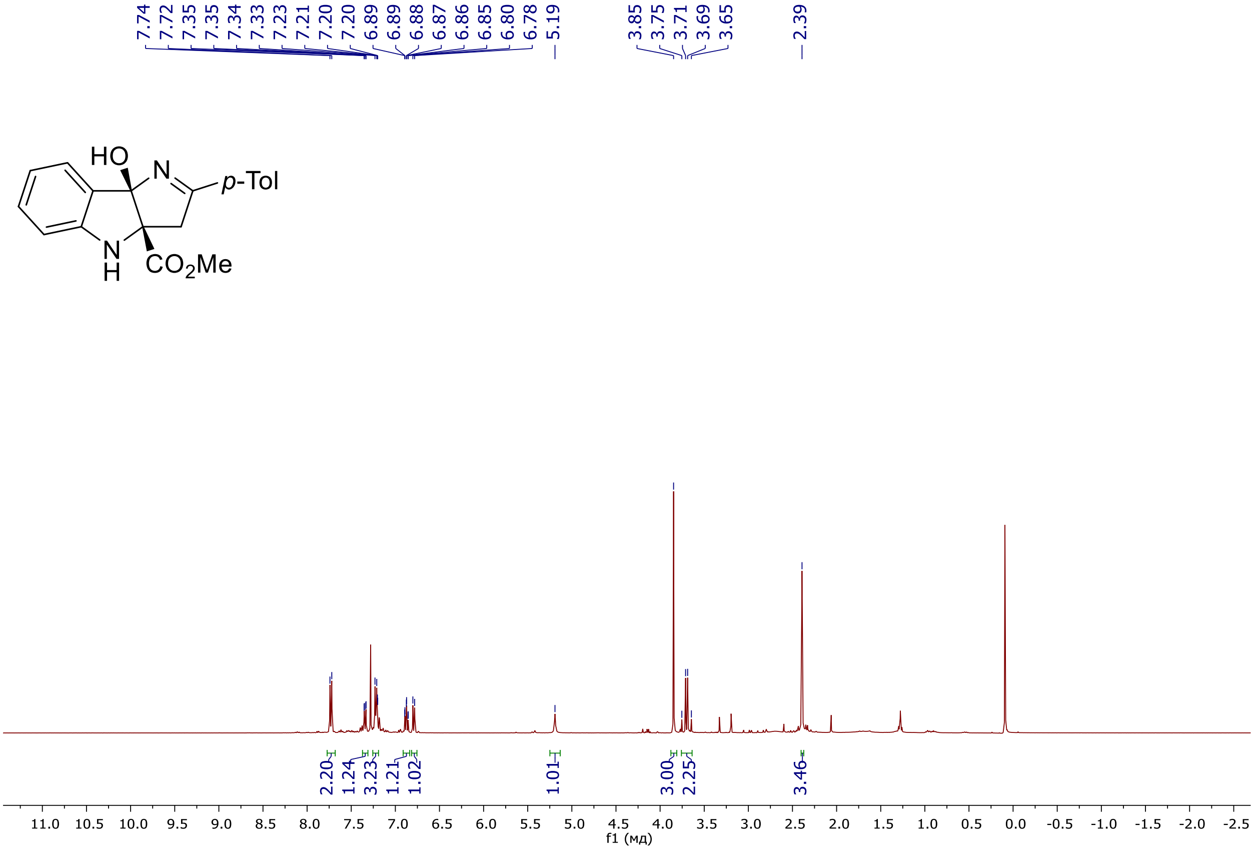

^{13}C{^{1}H} NMR spectrum of **11** (101 MHz, $CDCl_3$)

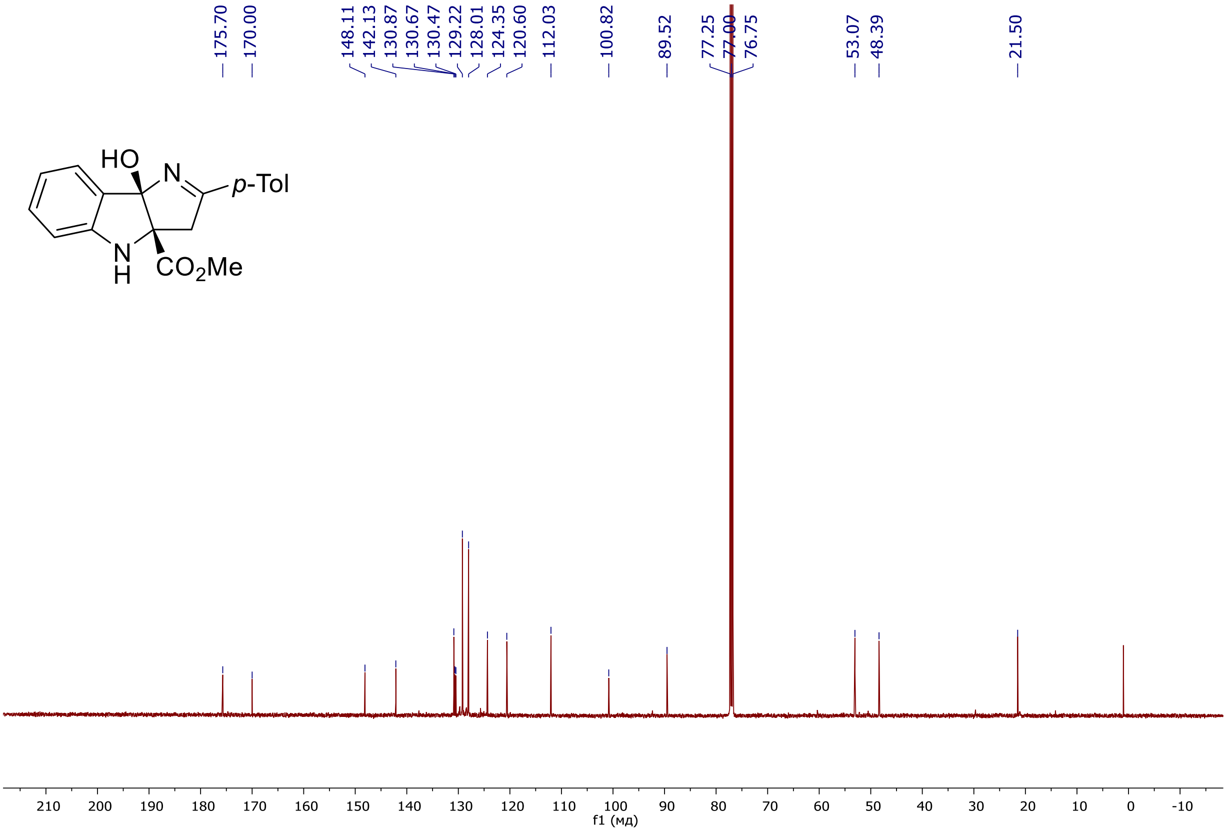

^{1}H NMR spectrum of **12** (400 MHz, $CDCl_3$)

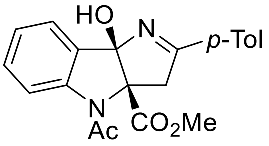

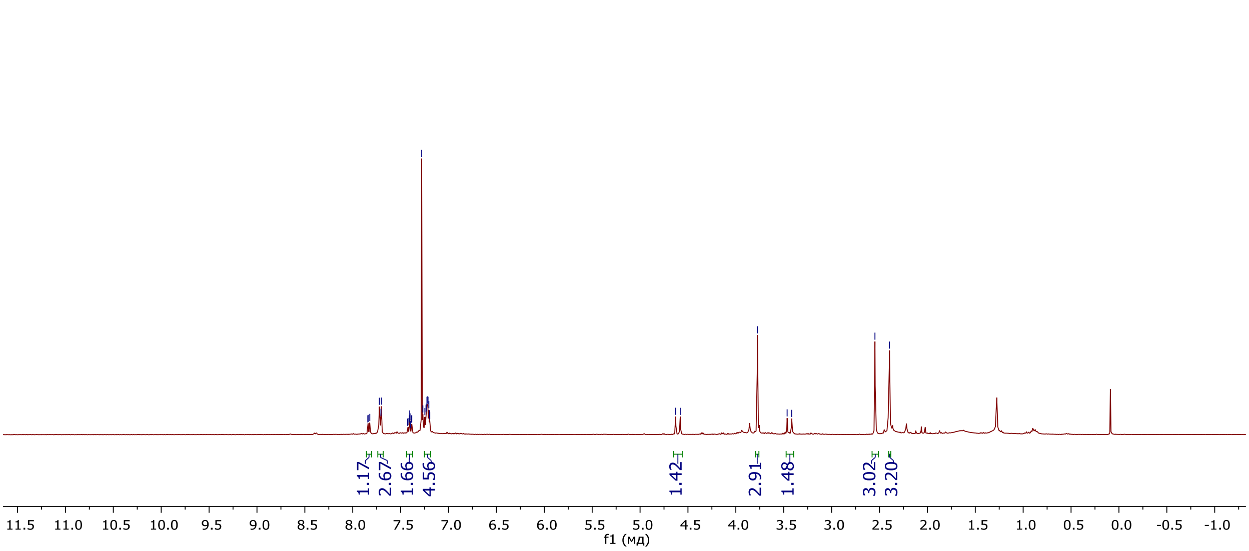

^{13}C{^{1}H} NMR spectrum of **12** (101 MHz, $CDCl_3$)

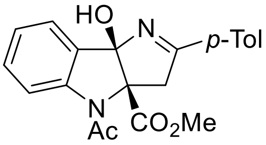

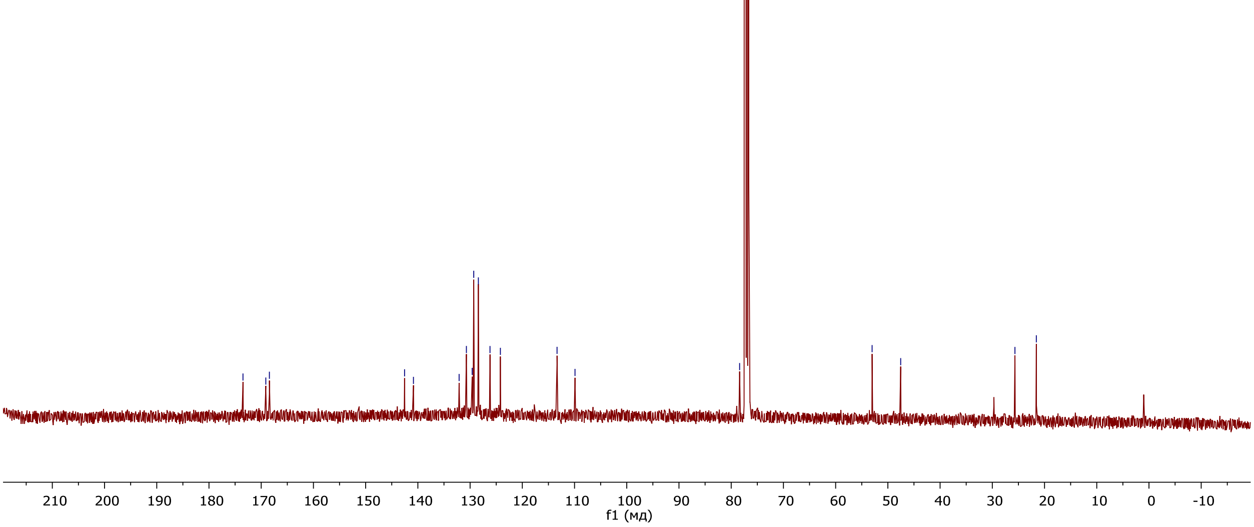

^{1}H NMR spectrum of **13** (400 MHz, $CDCl_3$)

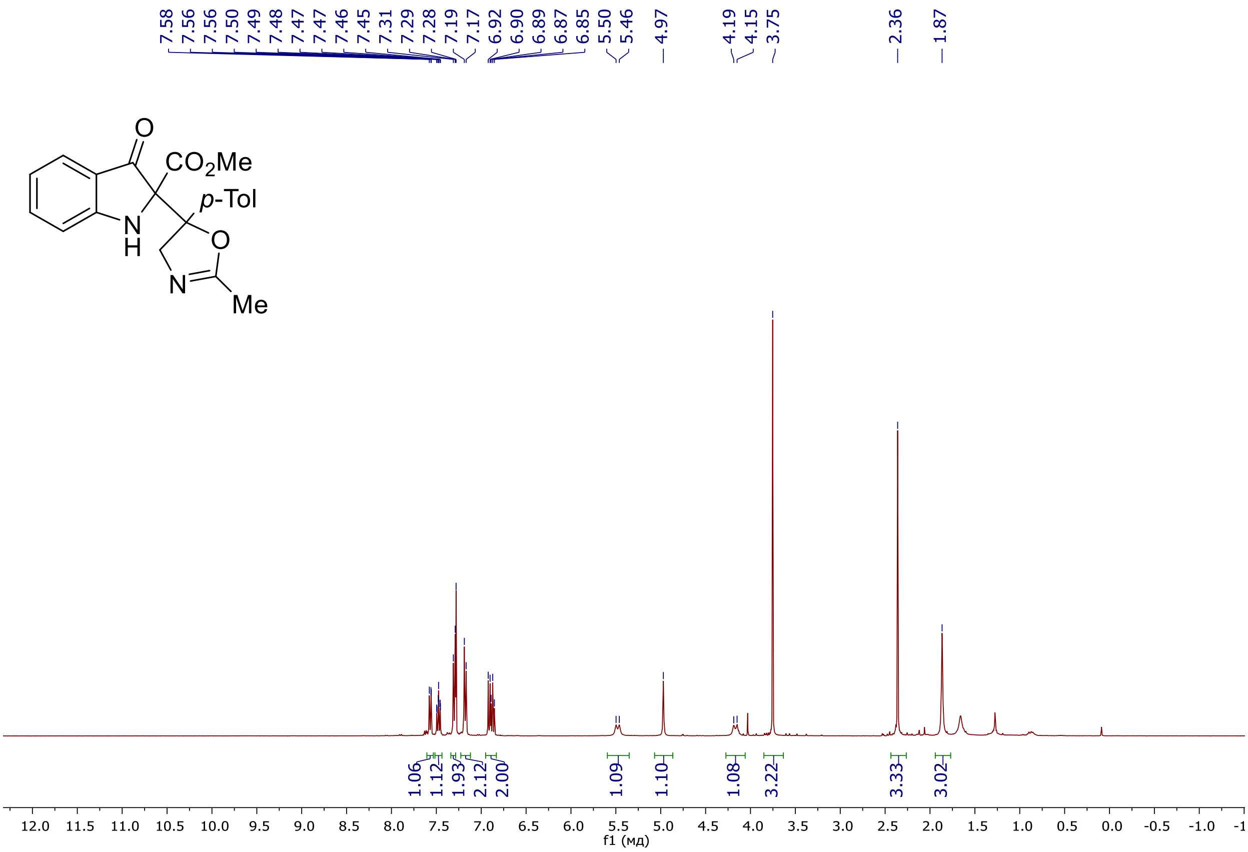

^{13}C{^{1}H} NMR spectrum of **13** (101 MHz, $CDCl_3$)

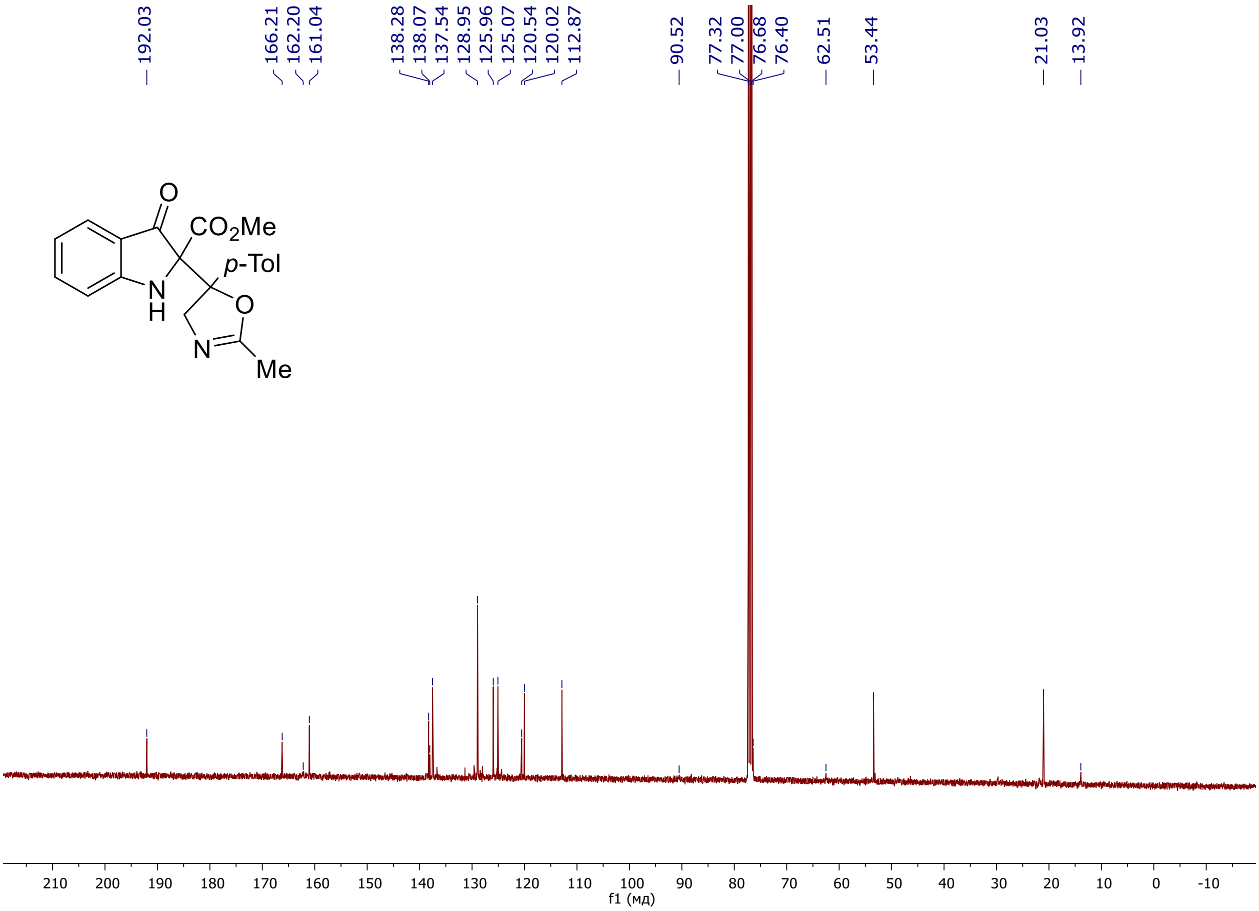

^{1}H NMR spectrum of **16** (400 MHz, $CDCl_3$)

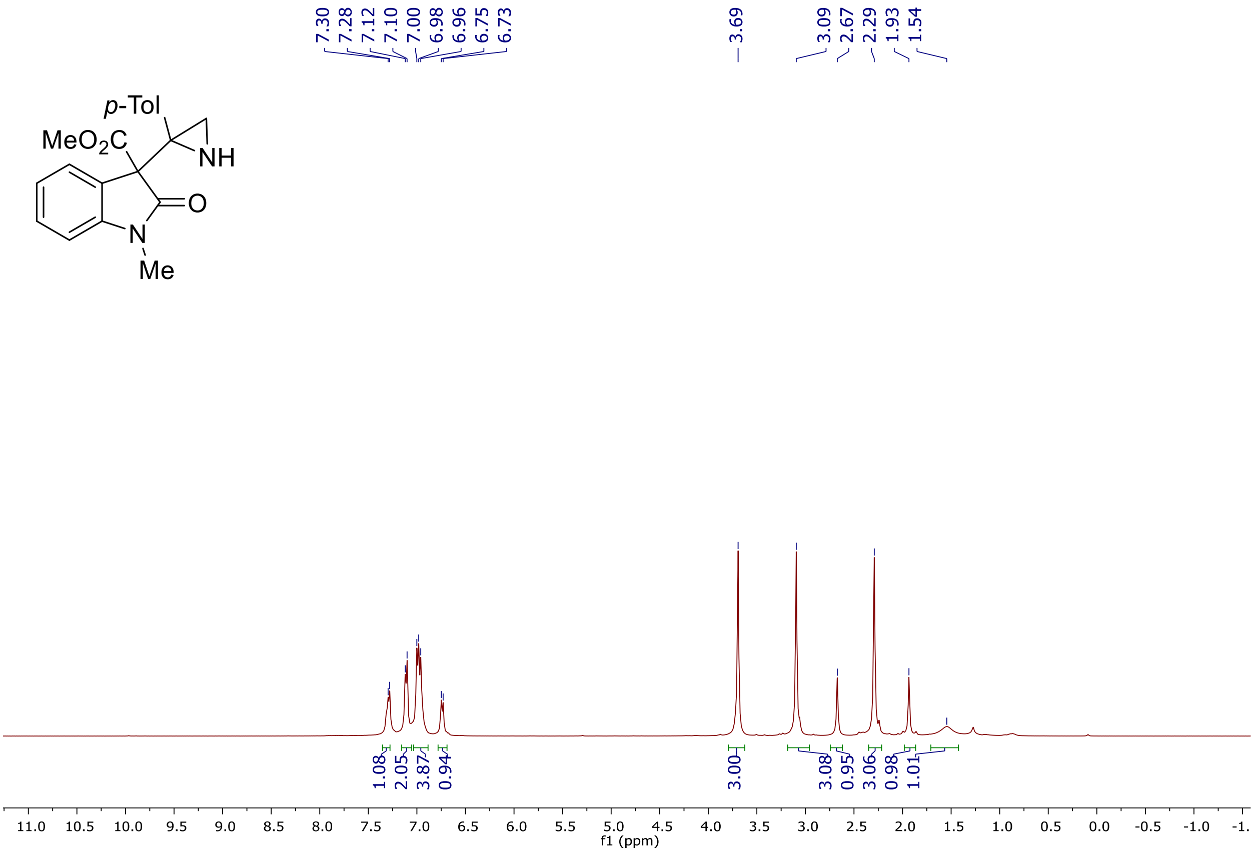

^{13}C{^{1}H} NMR spectrum of **16** (101 MHz, $CDCl_3$)

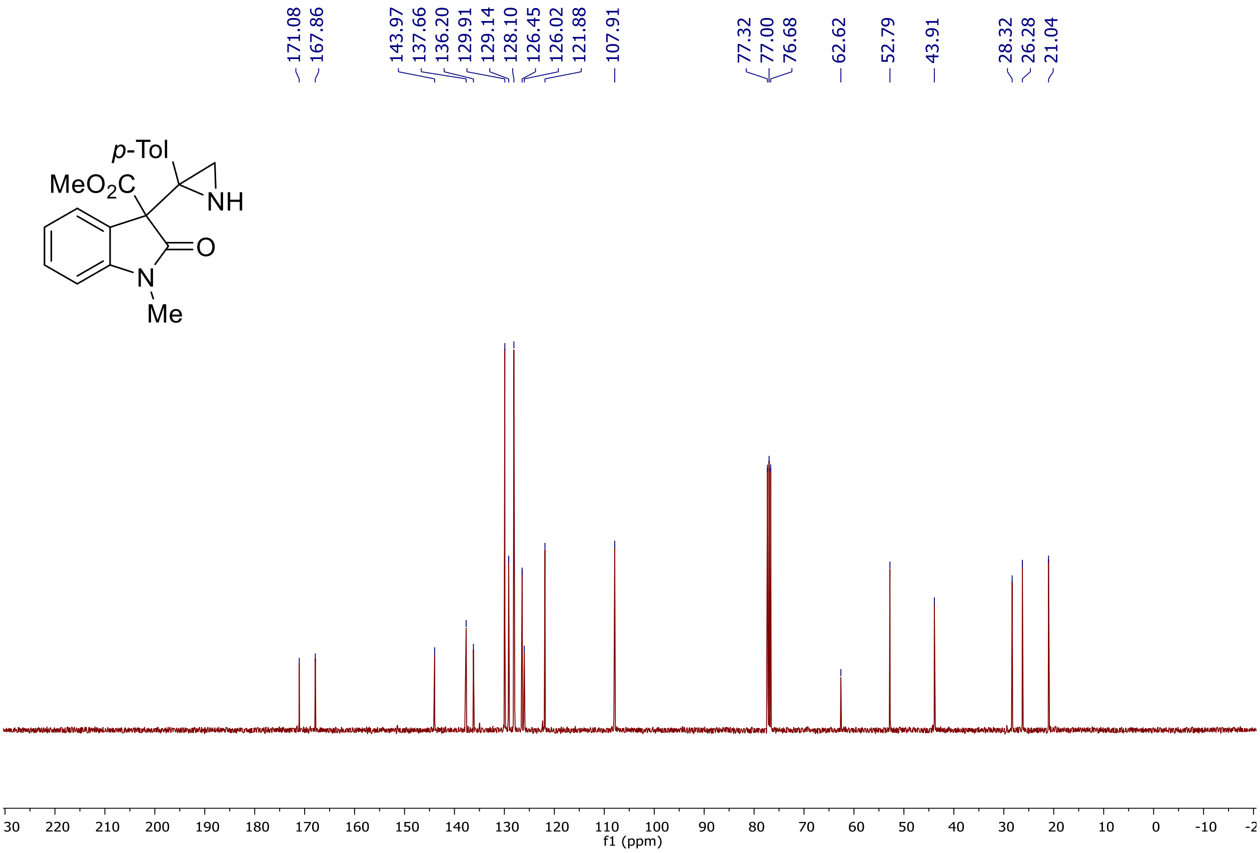

Supplement: File 1 — Full experimental details, characterization data and copies of NMR spectra for all new compounds. [file Beilstein_J_Org_Chem-21-1595-s001.pdf]
